# Efficient protein structure generation with sparse denoising models

**DOI:** 10.1038/s42256-025-01100-z

**Published:** 2025-09-24

**Authors:** Michael Jendrusch, Jan O. Korbel

**Affiliations:** 1 European Molecular Biology Laboratory (EMBL), Genome Biology Unit, Heidelberg, Germany; 2 Collaboration for Joint PhD Degree between EMBL and Heidelberg University, Faculty of Biosciences, Heidelberg, Germany

## Abstract

Proteins play diverse roles in all domains of life and are extensively harnessed as biomolecules in biotechnology, with applications spanning from fundamental research to biomedicine. Therefore, there is considerable interest in computationally designing proteins with specified properties. Protein structure generative models provide a means to design protein structures in a controllable manner and have been successfully applied to address various protein design tasks. Such models are paired with protein sequence and structure predictors to produce and select protein sequences for experimental testing. However, current protein structure generators face important limitations for proteins with more than 400 amino acids and require retraining for protein design tasks unseen during model training. To address the first issue, we introduce salad, a family of sparse all-atom denoising models for protein structure generation. Our models are smaller and faster than the state of the art and matching or improving design quality, successfully generating structures for protein lengths up to 1,000 amino acids. To address the second issue, we combine salad with structure editing, a sampling strategy for expanding the capability of protein denoising models to unseen tasks. We apply our approach to a variety of challenging protein design tasks, from generating protein scaffolds containing functional protein motifs (motif scaffolding) to designing proteins capable of adopting multiple distinct folds under different conditions (multi-state protein design), demonstrating the flexibility of salad and structure editing.

Computational protein design aims to generate protein sequences and three-dimensional structures with specified folds, functions and dynamics. Protein design tasks are varied, from designing proteins with a specified shape^1,2^ or symmetry^3–5^ and producing scaffolds for known functional motifs^2,6,7^ to designing potent binders for protein targets^1,8–10^ as well as proteins that can adopt multiple distinct folds under different conditions (multi-state design)^11–13^. Methods capable of solving these design tasks enable powerful applications in basic research and industry, for example, designing or optimizing enzymes^14–16^, antibodies^8^, vaccine scaffolds^17,18^ and biosensors^12^.

Protein generative models have recently been applied to solve many such protein design tasks^1,2,19,20^. Protein generation is fundamentally a multimodal generation problem, as proteins represent chains of amino acid residues, where each residue *i* carries an amino acid identity *s*_*i*_ and a set of atom coordinates **x**_*i*_. *s* is the protein’s sequence, and **x** is its structure. Designing proteins corresponds to sampling from the joint distribution of sequence and structure *p*(*s*, **x**|task) conditioned on a protein design task^21^. Many approaches to protein design decompose *p*(*s*, **x**|*task*) as *p*(*s*|**x**^bb^, task)*p*(**x**^bb^|task), where **x**^bb^ are the coordinates of the protein backbone atoms that are present in all amino acids^1,2,21^. This results in a sequential pipeline of backbone generation, followed by sequence design. However, there is no guarantee that proteins generated in this way will express, fold and function as designed in a living cell. Producing experimentally viable protein designs requires the generation and computational screening of many backbone–sequence pairs^1,20^. This computational design selection is enabled by the combination of sequence design models such as ProteinMPNN^20^, ChromaDesign^21^ or Frame2Seq^22^, as well as protein structure predictors such as AlphaFold 2 (ref. 23) and ESMFold^24^.

Designed structure–sequence pairs are deemed successful if their structure is predicted with high confidence and matches the initial design^1^. The prediction confidence is measured in terms of the predicted local distance difference test (pLDDT)^25^ and the predicted aligned error (pAE)^23^ of the structure predictor used in the pipeline. Designed and predicted structures are considered to be in agreement if their root mean square deviation (RMSD), which measures the average distance between superimposed atoms, is low^1^. RMSD measures consistency between design and prediction and is generally referred to as self-consistent RMSD (scRMSD)^1,26^. A common choice of success criteria is scRMSD < 2 Å and pLDDT > 70 for ESMFold^2^ or pLDDT > 80 for AlphaFold 2, which have been shown to produce experimentally viable proteins^1,10,27,28^. This measure allows to compare different protein structure generators in terms of their designability, the fraction of generated structures for which at least one designed sequence meets the criteria for success. In addition to designability, models can be compared in terms of the diversity of their generations, as well as their novelty compared with the training set. Diversity can be measured in terms of the template modelling (TM) score^2,29^ within a set of generated structures. By contrast, novelty uses the TM-score to measure the dissimilarity between a model’s generated structures and its training set^2^. Together, these metrics characterize the performance of protein structure generators.

Current approaches to protein design combine multiple different methods for protein backbone generation. Knowledge-based design coupled with Rosetta^30^ plays an important role, especially for complex protein design tasks, such as enzyme design^15,16^, multi-state protein design^11,12^, and protein design with strong geometric and sequence constraints^3,4^. These methods are supported by machine learning models, which are capable of solving simpler design tasks without relying on Rosetta or manual design. Protein structure hallucination methods^10,27,31–34^ invert structure predictors using search or gradient descent to generate sequences with high-confidence predicted structures. These sequences are often adversarial and, therefore, discarded in favour of ProteinMPNN sequence designs^10,27,31^. Protein denoising diffusion probabilistic models^35^ iteratively generate proteins from random noise by learning to remove noise from corrupted protein sequences^13,36^ or structures^1,2,21,37,38^. Diffusion models have a runtime advantage over hallucination-based methods as they do not require optimization over a structure predictor^1,27^. Protein diffusion models have recently been applied to solve various protein design tasks from unconstrained de novo protein design^1,2,21^ to protein binders and complexes^1,21^.

Although current protein diffusion models have shown impressive performance for small protein generation, their performance deteriorates with protein sequence length *N*^1,2,21,37^, limiting their usefulness for designing large and complex proteins. The majority of protein diffusion models use model architectures derived from protein structure predictors^23,39^. Notable exceptions include Chroma^21^, Protpardelle^40^ and ProteinSGM^41^. Models based on structure predictors use residue-pair features and pair attention mechanisms, which result in *O*(*N*^3^) complexity, with pair features introducing a lower complexity bound of *O*(*N*^2^) and pair attention mechanisms increasing this complexity to *O*(*N*^3^) (refs. 23,39). Along with decreased runtime performance, these models also experience a drop in designability with increasing *N*. Although recent work on Proteus^37^ and Proteina^42^ has improved designability for proteins up to 800 residues long, no protein structure diffusion model has reached the designability of hallucination-based approaches beyond that length^27^. However, protein backbone hallucination suffers from long runtimes per design at these lengths^27^.

This greatly reduces throughput and limits the applicability of backbone hallucination to large protein design tasks with lower per-design success rates compared with unconditional monomer design.

Another issue with current protein structure diffusion models is the need for additional training to solve specific protein design tasks. RFdiffusion and Genie are separately trained with protein motif conditioning to scaffold functional protein motifs^1,2^. Although Chroma’s conditioners allow for training-free adaptation of the model for different tasks, implementing new conditioners is not straightforward and requires the development of custom energy functions^21^. Thus, there is a substantial barrier to applying existing diffusion models to novel design tasks.

To address these issues, we introduce salad (sparse all-atom denoising), a family of efficient protein generative models with sub-quadratic complexity. We train our models with a denoising diffusion objective^1,35^ to remove noise from corrupted protein backbones. Starting from a sparse transformer architecture^21,43,44^, we investigate the impact of different model features and noise schedules on the designability and diversity of generated proteins. We find that our models are capable of generating diverse and designable backbones for proteins up to 1,000 residues long. salad matches or outperforms state-of-the-art diffusion models^1,2,42^ in terms of designability and drastically reducing runtime and parameter count. We combine salad with structure editing, a modified sampling algorithm for protein structure diffusion models. By editing the input noise and output of the model, we can enforce arbitrary structural constraints without the need for model retraining. This enables rapid prototyping of protein design tasks unseen during training. For example, we can symmetrize both model input and output to generate symmetric proteins, or replace residue coordinates with the coordinates of a protein structural motif to embed that motif in the generated structure. Structure editing allows us to tackle a variety of protein design tasks, generating designable backbones with specified shapes^21^, scaffolds for functional protein motifs^1,2,6^, repeat proteins^3,4,13^ and multi-state proteins that adopt distinct folds when cleaved^13^. In this way, salad provides an efficient plug-and-play replacement for other backbone generators in existing protein design pipelines, allowing fine-grained control via structure editing and enabling efficient design of large proteins.

## Results

### Sparse protein model architecture

**Fig. 1:**
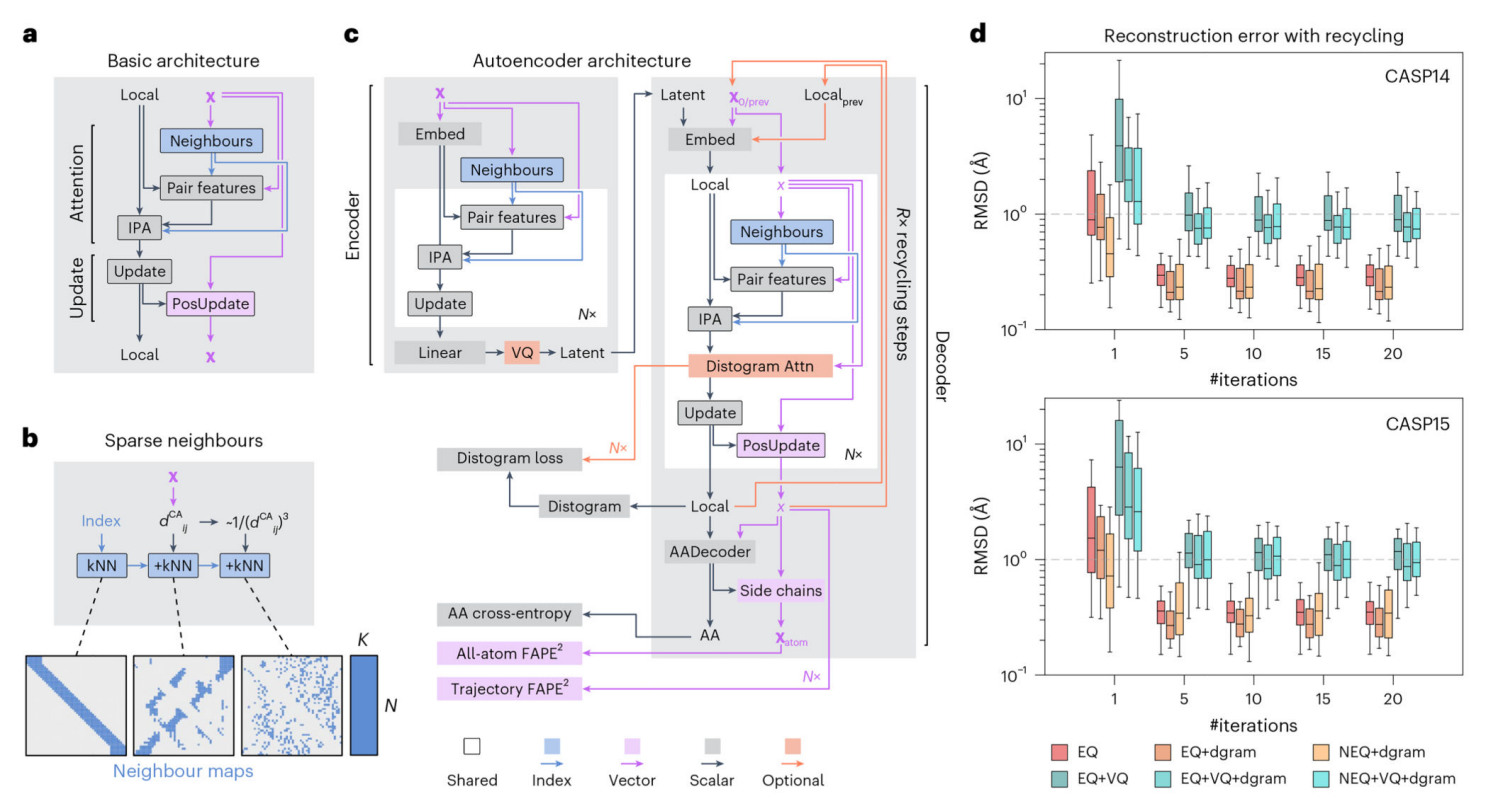
Sparse model architecture. **a**, Schematic of the basic block of our architecture. **b**, Schematic of neighbour selection using residue index, nearest and random neighbours. **c**, Architecture of our sparse protein autoencoder. **d**, Sparse model performance on an autoencoding task. Box plots of scRMSD between the ground-truth and decoded structures for multiple sparse architectures: equivariant (EQ) and non-equivariant (NEQ). Optionally, we use predicted distograms for neighbour selection (dgram) and vector quantization (VQ). Measures of reconstruction performance are shown per number of model recycling iterations. The dotted line indicates the threshold of 1 Å for reconstruction at atomic precision. The box centre line indicates the median, the boundaries indicate the 1st and 3rd quartiles, and whiskers show the 1st or 3rd quartile + 1.5 times the interquartile range based on *n* = 34 CASP14 test structures and *n* = 45 CASP15 test structures.

We base our model architecture on the current best practices for transformer models. We use layer norm pre-normalization to increase the training stability^45^ and generalized Gaussian linear units (GeGLU) feed-forward layers that were found to improve model performance for transformer models^46^. We replace standard multi-head attention by invariant point attention (IPA) introduced by AlphaFold 2 (ref. 23) as an easy-to-implement SE(3)-equivariant self-attention layer. To improve the runtime complexity of protein structure generation, we limit the attention operation to a sparse set of neighbours for each amino acid residue. A schematic of the basic block of this architecture is shown in Fig. 1a. Each block in our model takes as input a set of amino acid features local_*i*_ and position features **x**_*i*_. These are fed into a sparse version of IPA^23^. Instead of computing the full attention matrix and pair features, we first construct a set of neighbours for each amino acid (Fig. 1b). Each residue only computes pair features and attention weights for its set of neighbours. This procedure reduces attention complexity from *O*(*N*^2^) to *O*(*N* · *K*), where *N* is the number of residues and *K* is the number of neighbours. In contrast to other protein generative models^1,2,21^, our model does not use persistent pair features with pair attention or triangle multiplication^23^, which would increase complexity to *O*(*N*^3^). We also do not use explicit amino acid frame features^23^ that are updated in each block. Instead, our models directly update atom positions and recompute frame information when required to ensure equivariance. We reuse this basic block architecture (Fig. 1a) across all models in this work.

As the first step to see if our sparse attention architecture can model protein structures, we trained a family of models as autoencoders on proteins in the Protein Data Bank (PDB)^47^ (Fig. 1c). We encoded protein structures using a single basic block with 32 nearest neighbours in Euclidean space per residue. We then optionally applied vector quantization (+VQ) to the resulting latent representation^48^ and decoded it using a six-block decoder with recycling^23^, reusing the previous iteration’s residue coordinates and local_*i*_ features. VQ regularizes the latent space of the encoder and quantizes it into a set of discrete tokens for each residue^48,49^. This enables using the learned representation to train sequence generative models on structure-based data^49^. We tested both SE(3) equivariant and non-equivariant sparse transformers to check if there are any benefits to equivariance for the autoencoding task. Additionally, we investigated different neighbour-selection schemes. By default, we selected nearest neighbours based on distance along the protein chain and Euclidean distance between residues (Fig. 1b and Supplementary Algorithm 3). As Euclidean distances would be uninformative at the beginning of the decoding process, we optionally selected additional neighbours using average residue-pair distances predicted from local_*i*_ by learning to predict a distogram^23^ in each block (+dgram, Fig. 1c (distogram attn.); also see the ‘Structure autoencoder models’ section and Supplementary Algorithms 7 and 8).

Evaluating these models on the CASP14 and CASP15 monomer test sets^50,51^ resulted in all models reaching <1 Å reconstruction accuracy after fewer than ten recycling iterations, regardless of equivariance and distogram-based neighbours (Fig. 1d). This indicates that our sparse attention architecture is expressive enough to model protein structures. Although there is a large difference in model performance between different architectures at one recycling iteration, this difference decreases with the number of iterations. We decided to keep the simplest version of our architecture using equivariant features without distogram neighbours for the rest of this work.

### Edited denoising protein models

**Fig. 2:**
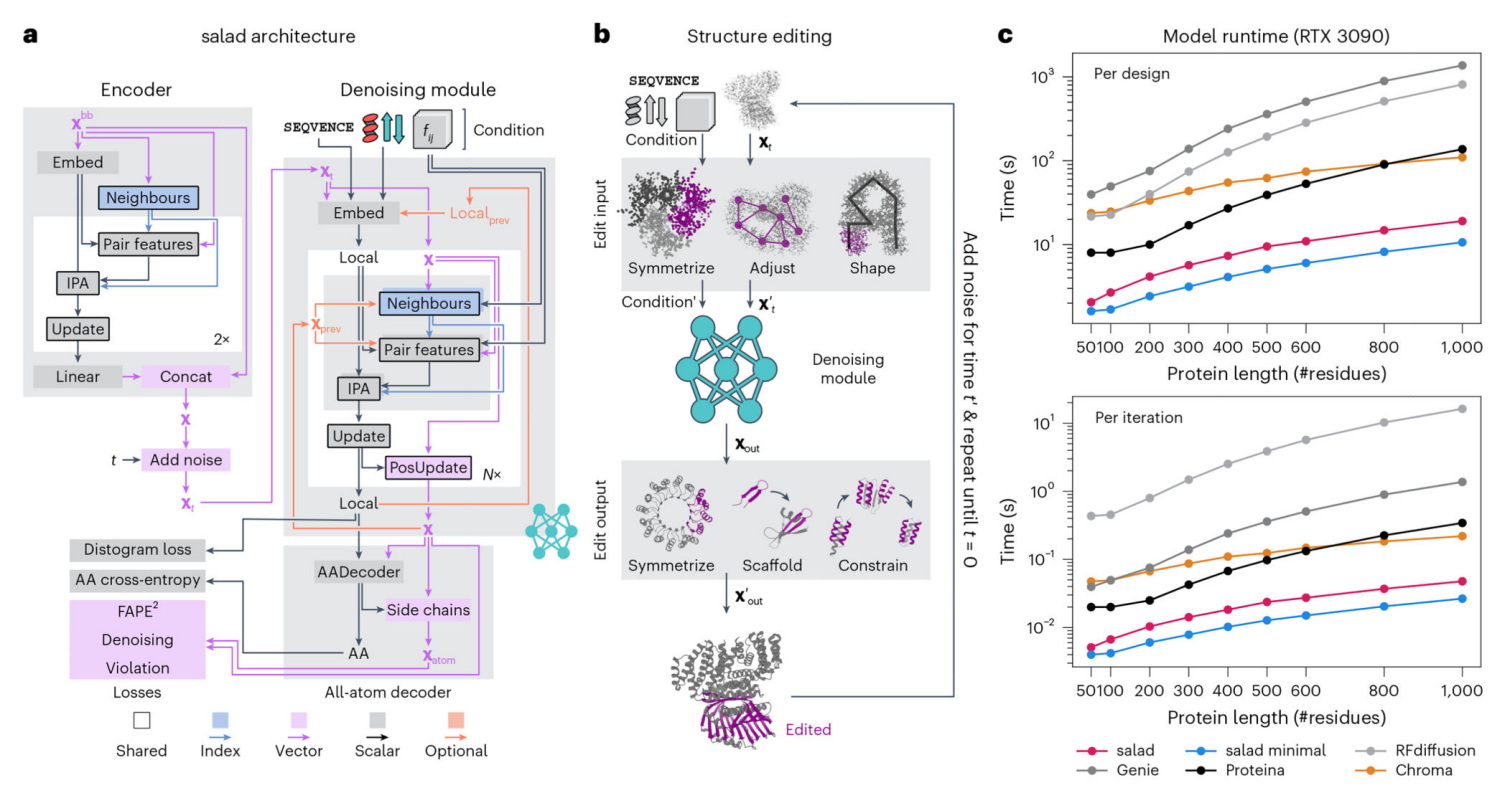
Denoising model architecture and runtime. **a**, Schematic of the salad architecture. **b**, Sampling process of a salad model with structure editing, with example applications of input and output editing. **c**, Average runtimes on a single RTX 3090 GPU by number of amino acid residues of sparse diffusion models (full pair features and minimal pair features) compared with RFdiffusion, Genie 2, Chroma and Proteina. Runtimes are reported per designed structure, using the default number of denoising iterations for each model (salad, 400; Chroma, 500; Genie 2, 1,000; RFdiffusion, 50; Proteina, 400) as well as the time per iteration. Mean time is based on *n* = 10 generated backbones per size, and the generation time of the first backbone per size is discarded to exclude model compilation time from the average.

After ensuring that our sparse models are suitable for reconstructing protein structures, we modified our architecture for generative modelling. Our models operate on protein structures containing the backbone atoms (N, CA, C and O), an idealized beta-carbon (CB) and additional learned pseudo-atoms (Fig. 2a; see the ‘Model architecture’ section). We trained our models to denoise noisy structures **x**_*t*_ ~ *p*(**x**_*t*_|**x**_0_) and to recover the original structure **x**_0_, resulting in a denoising diffusion probabilistic model loss ${ℒ}_{t}=E_{x_{t}\sim p\left({\text{x}}_{t}|{\text{x}}_{0}\right)}\left[∥f_{\theta }\left({\text{x}}_{t}\right)-{\text{x}}_{0}{∥}^{2}\right]$ ref. 35). In addition to recovering **x**_0_, we introduced auxiliary losses to also predict an amino acid sequence and side-chain atom positions (Fig. 2a; also see the ‘Denoising model loss’ section). As our models generate all-atom structures, we refer to them as sparse all-atom denoising (salad) models throughout this work.

At inference, we can use our models to generate protein backbones by progressively denoising a pure noise structure **x**_1_. Given a noisy structure **x**_*t*_, we can use the model to predict an estimate *f*_*θ*_(**x**_*t*_) of the denoised structure **x**_0_. Reapplying noise at a lower diffusion time *t*′ results in a structure **x**_*t*′_ ~ *p*(**x**_*t*′_|*f*_*θ*_(**x**_*t*_)) (Fig. 2b). Repeating this process eventually results in a generated structure **x**. To enforce the structural properties of generated backbones directly in the denoising process, we introduce editing functions edit_input and edit_output, which modify the input and output of the denoising model, respectively (Fig. 2b). This results in a generative process:

(1) $$x_{t^{\prime }}\sim p\left(x_{t^{\prime }}|\text{edit}\_\text{output}\left(f_{\theta }\left(\text{edit}\_\text{input}\;\left(x_{t}\right)\right)\right)\right).$$

Designing suitable editing functions allows us to adapt our models to various tasks, from motif scaffolding to multi-state protein design, without having to re-train our models.

As we are using sparse models for the sake of runtime efficiency, we compare the runtime performance of our models to state-of-the-art protein diffusion models (RFdiffusion, Genie 2, Chroma and Proteina). To see how far we can push the model runtime, we tested both full and lightweight versions of our model. For each model, we generated ten protein backbones per length (50–1,000 residues) on a single NVIDIA RTX 3090 GPU (Fig. 2c). salad outperformed all other models in terms of both time per design at the default number of diffusion steps and time per model iteration. Compared with the fastest non-salad model (Proteina), our models reached up to 7× speed-ups, and outperformed RFdiffusion by up to two orders of magnitude on large proteins. Indeed, generating a 1,000-residue protein structure using salad on a single NVIDIA RTX 3090 GPU takes only 19 s on average, whereas RFdiffusion takes over 10 min. In addition, our models use fewer parameters than comparable protein structure generators (Extended Data Table 1). This suggests that we have indeed reached our primary goal of implementing a runtime- and parameter-efficient protein generative model.

### Sparse models generate diverse and designable protein structures

**Fig. 3:**
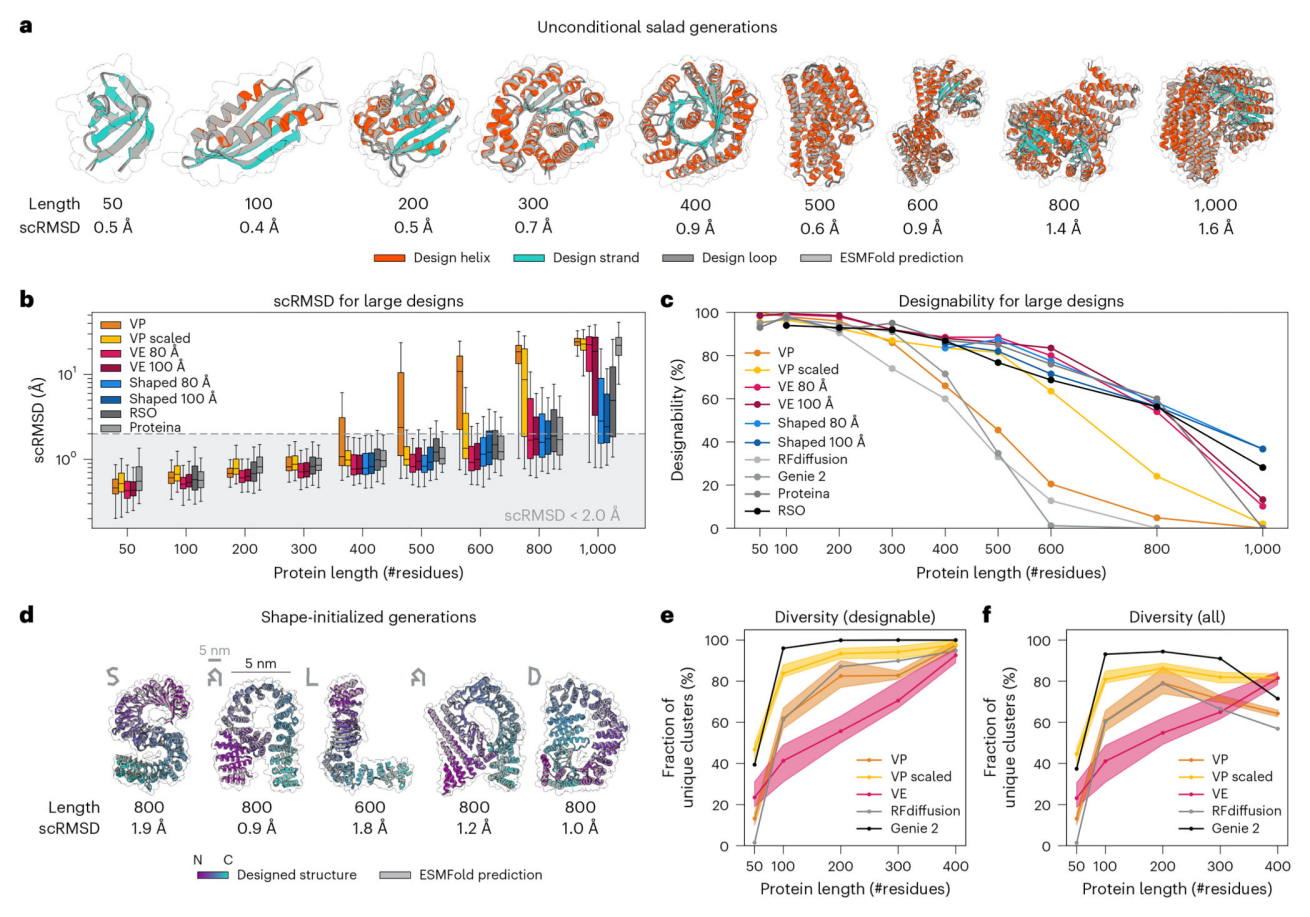
Unconditional structure generation with salad models. **a**, Example unconditional generations using salad, ranging from 50 to 1,000 residues (coloured by secondary structure; loop, grey; helix, red; strand, blue) and their ESMFold-predicted structures (light grey). The scRMSD between generation and ESMFold prediction is listed underneath each structure. **b**, scRMSD values using ESMFold for designed structures from 50 to 1,000 residues for salad, RFdiffusion^1^, Genie 2 (ref. 2), Proteina^42^ and RSO^27^. The region of successful designs with scRMSD < 2.0 Å is marked in light grey. The box centre line indicates the median scRMSD, the boundaries indicate the 1st and 3rd quartiles, and whiskers show the 1st or 3rd quartile + 1.5 times the interquartile range. **c**, Designability of generated structures by protein length from 50 to 1,000 residues. The shown RSO values are from ref. 27. Data in **b** and **c** are based on *n* = 200 generated backbones per length. **d**, Generations (coloured by residue index) and ESMFold predictions (light grey) for large protein structures generated using VE denoising starting from letter-shaped noise. Each structure is reported with its scRMSD between generation and ESMFold prediction. The grey letter shape in the top left corresponds to the shape of the noise the proteins were generated from. Scale bar (grey), 5 nm. The black bar corresponds to the same distance in the depicted protein structures. **e**,**f**, Model diversity computed as the fraction of designable clusters over designable structures (**e**) or all the generated structures (**f**). Clusters are generated using single-linkage clustering by pairwise TM-score with a cut-off of TM-score ≥ 0.6. Mean diversity across *n* = 10 random samples of 100 structures from 200 generated structures. Error bars indicate the minimum and maximum diversities across *n* = 10 samples. Area between the maximum and minimum sampled diversity is shaded.

Although a flexible sampler and good runtime performance are important properties of our models, we need to assess model performance in terms of the quality of the generated backbones. To compare salad model performance to state-of-the-art diffusion models and hallucination-based approaches, we generated 200 backbones each for proteins with sizes of 50 to 1,000 residues (50, 100–600 in increments of 100, 800 and 1,000). For each backbone, we designed eight sequences using ProteinMPNN and predicted their structures with ESMFold. Following current best practices^2,27^, we computed designability as the percentage of structures, reaching an RMSD between design and predicted structure (scRMSD) < 2 Å and pLDDT > 70 for the best designed sequence (Fig. 3a). We assessed the impact of different noise distributions on protein structure generation by comparing model performance with both variance-preserving (VP) and variance-expanding (VE) noise with different standard deviations (80 Å and 100 Å, respectively). In addition, we include models trained with protein-length-dependent variance VP noise (VP scaled), as the variance of atom positions in protein backbones increases with the number of residues (Extended Data Fig. 4a). We compare the results of our models (VP, VP scaled and VE) with RFdiffusion^1^, Genie 2 (ref. 2) and Proteina^42^, as well as results from relaxed sequence optimization (RSO)—the state-of-the-art hallucination-based method for protein design^27^—using the same evaluation approach for all methods.

Our models are able to generate designable backbones for a variety of protein lengths from 50 to 1,000 residues (Fig. 3a and Extended Data Fig. 2). The generated structures show low scRMSD, high scTM/pLDDT and diverse secondary structures that include both all-helix and all-strand topologies (Fig. 3a,b and Extended Data Figs. 3 and 5a,b). In the range from 50 to 400 residues, our VP model reaches comparable designability to previous VP models (Genie 2 and RFdiffusion), outperforming RFdiffusion and slightly underpeforming compared with Genie 2, which was trained on a much larger dataset^2^ (Fig. 3c). At 400 residues, all VP models show a sharp increase in scRMSD accompanied by a decrease in designability (Fig. 3b,c). Although neither Genie 2 nor RFdiffusion produce any designable structures with 800 residues, salad VP still produces 4.8% designable structures at that size. We suspect that the decrease in designability with residue count is caused by VP diffusion models generating highly compact backbones (Extended Data Fig. 4a). We find that such backbones require a high fraction of glycine and alanine residues to avoid clashes (Extended Data Fig. 4b,c), which might decrease designability. A likely cause of this is the fixed variance of the VP diffusion process, which requires the model to reduce amino acid distances at small protein sizes, but increase amino acid distances at large protein sizes (Extended Data Fig. 4d). If the model trains predominantly on small proteins, this discrepancy might result in the observed compact backbones for larger proteins at inference. This issue with protein diffusion models is anecdotally known to the protein design community^52^.

By contrast, VP-scaled and VE models do not experience increases in scRMSD at the 400-residue threshold. VP-scaled models maintain median scRMSD < 2 Å for proteins of up to 600 residues, whereas VE models maintain this value for proteins up to 800 residues in length (Fig. 3b). This is mirrored by designability, where both VP-scaled and VE models outperform all VP models at protein lengths above 300 residues. However, neither VP-scaled nor VE models can maintain high designability for generated backbones of length 1,000, where both types of model drop below 20%. We hypothesized that this decrease in designability is due to the models being unable to properly model the global structure of large proteins. As large proteins generally consist of multiple domains in which residues of a single domain are close in space^53^, we tested if VE models initialized from noise shaped in a similar way would result in lower scRMSD and greater designability for large proteins. Instead of using normal-distributed noise centred on the coordinate origin, we first sample a set of centres and then add normal-distributed noise (with standard deviation of 80 Å or 100 Å) for 200 residues to each of these centres. At every subsequent denoising step, we use standard VE noise. Using this shaped-noise initialization leads to decreased scRMSD and increased designability for large proteins, reaching a designability of up to 36.7% for 1,000 amino acid proteins (Fig. 3c). This way, shaped noise matches or improves on the designability of RSO^27^ and Proteina^42^, the current state-of-the-art hallucination- and diffusion-based approaches to large protein design, respectively (Fig. 3b,c). Strikingly, salad with shaped noise produces designable 1,000-residue proteins with only ~8M parameters, compared with concurrent work introducing Proteina, which uses 200M parameters (Extended Data Table 1) and does not result in any designable structures at 1,000 residues (Fig. 3b,c).

**Extended Data Fig. 1:**
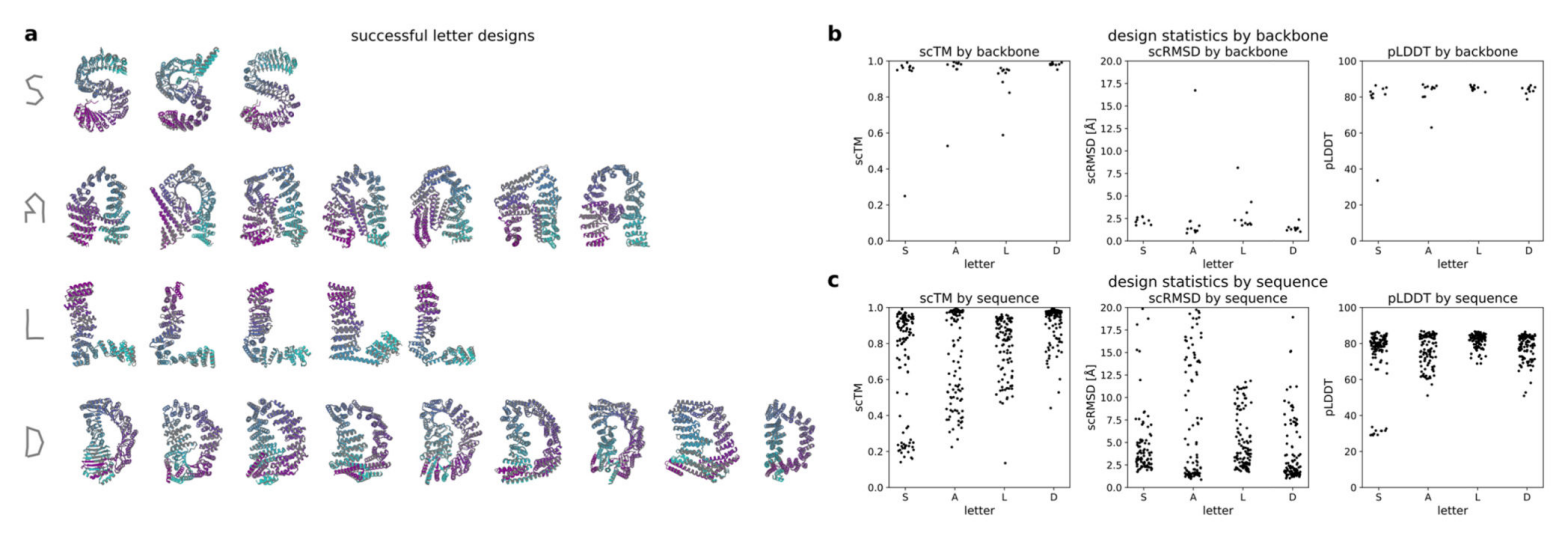
Letter shape generation. (**a**) Successfully designed letter shapes for letters (S, A, L, D). The designed structure (grey) is overlaid with the best ESMfold prediction (coloured by residue index) out of 10 ProteinMPNN sequences. (**b, c**) scRMSD, scTM and pLDDT for each backbone (**b**, n = 10) or designed sequence (**c**, n = 110, 1 salad sequence and 10 ProteinMPNN sequences per backbone) across all letter designs.

As we can use shaped noise to generate large proteins with VE models, we investigated if we could control the shape of the generated backbones by directly specifying the positions of noise centres used for shaping. By sampling the initial noise centred on letter shapes, we were able to generate designable structures spelling out the name of our framework (Fig. 3d). In contrast to previous work on shape-conditioned protein design using the Chroma model^21^, our approach does not require an additional shape conditioner and results in designs with low scRMSD and high pLDDT (Fig. 3d). In terms of standard designability criteria using ESMFold (scRMSD < 2 Å, pLDDT > 70), 55% of the letters generated by our models are designable, whereas up to 92.5% of the letters are re-foldable according to the criteria used for Chroma (scTM > 0.7)^21^ (Extended Data Fig. 1). This indicates that our models can be used to generate designable backbones even on challenging out-of-distribution design tasks.

In addition to designability, we measure the diversity of protein backbones generated by our models. Lin et al.^2^ previously quantified the diversity of generated backbones by performing hierarchical clustering with single linkage on designable structures, using a TM-score threshold of 0.6 to define distinct clusters. Diversity is then computed as the fraction of designable clusters in all generated backbones: ${\text{diversity}}_{all}=\frac{\;\#\text{clusters}}{\#\text{all}}$ (ref. 2). This diversity measure implicitly includes backbone designability, as a lower number of designable backbones results in a lower number of clusters. A method trading off designability for increased diversity would, therefore, result in a low diversity_all_ score. To disentangle diversity and designability, we decompose this diversity score as follows.

(2) $${\text{diversity}}_{all}=\frac{\#\text{clusters}}{\#\text{all}}=\underset{{\text{diversity}}_{designable}}{\underset{︸}{\frac{\#\text{clusters}}{\#\text{designable}}}}\cdot \underset{\text{designability}}{\underset{︸}{\frac{\#\text{designable}}{\#\text{all}}}}$$

When computing diversity_designable_, only the diversity of designable structures is taken into account and diversity is not deflated by low designability. We argue that this is a more meaningful measure of diversity as only designable structures are used for protein design in the end.

We compare our models with RFdiffusion^1^ and Genie 2 (ref. 2). For proteins of length 50–400 residues, we take random samples of 100 generated structures and compute both diversity_all_ and diversity_designable_ for each sample. To quantify the spread of diversity across samples, we show the median as well as the minimum and maximum diversities over ten samples (Fig. 3e,f). Our VP model achieves similar diversity to RFdiffusion for both diversity_designable_ and diversity_all_, whereas the VP-scaled model outperforms RFdiffusion on both metrics and approaches the diversity of Genie 2, outperforming it in terms of diversity_all_ for 400-residue proteins. Our VE model shows reduced diversity at small protein sizes, but shows comparable diversity_designable_ to RFdiffusion on 400-residue proteins and outperforms both Genie 2 and RFdiffusion in terms of diversity_all_ for this protein length. This indicates that designable 400-residue structures generated by our non-VP models are comparably diverse to those generated using Genie 2. Their increased diversity_all_ can be attributed to their improved designability (Fig. 3c). We, therefore, argue that diversity_designable_ is a more meaningful measure of diversity as it is not inflated by changes in designability. Although our models slightly underperform Genie 2 in terms of diversity, we note that Genie 2 was trained on AlphaFold DB^54^—a larger and more diverse dataset.

### Random secondary structure conditioning maximizes diversity

**Fig. 4:**
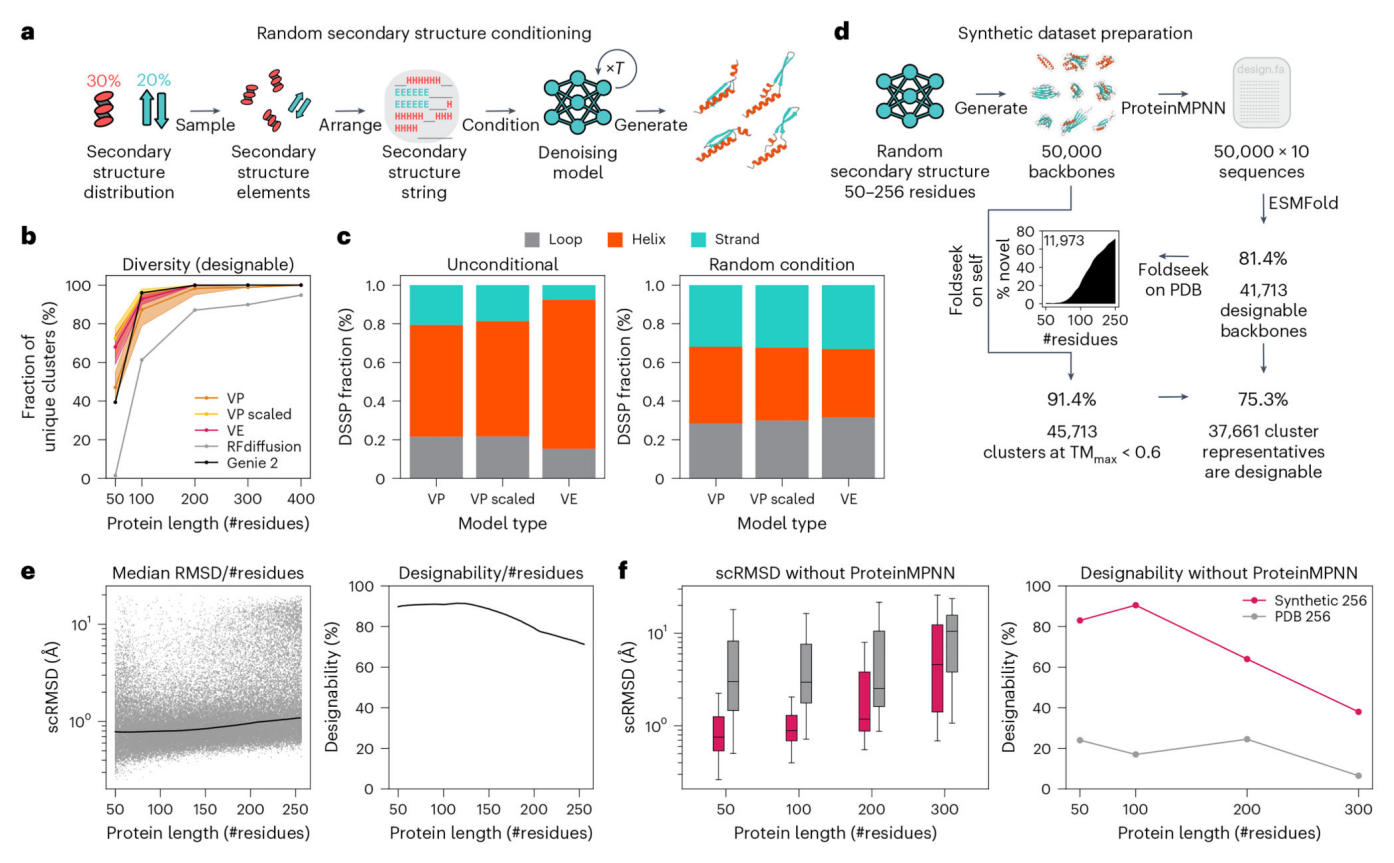
Random secondary structure conditioning maximizes diversity at the cost of designability. **a**, Schematic of our random secondary structure sampling procedure. **b**, Diversity of designable structures generated using random secondary structure conditioning for 50–400 residues, compared with the diversity of designable structures generated using RFdiffusion and Genie 2. Mean diversity across *n* = 10 random samples of 100 backbones from 200 generated backbones. Error bars correspond to minimum and maximum diversities across these *n* = 10 samples. Area between the maximum and minimum sampled diversity is shaded. **c**, Secondary structure distribution for our models using no conditioning (left) or random secondary structure conditioning (right). **d**, Overview of diverse synthetic dataset generation using random secondary structure conditioning. **e**, Left: scatter plot of ESMFold scRMSD for all 50,000 generated structures. The line indicates the median scRMSD within a length window of 100 residues. Right: designability of the generated structures in the synthetic dataset computed for a length window of 100 amino acids. **f**, Single-shot performance of diffusion models trained on the synthetic dataset (synthetic 256) compared with the subset of proteins of length of <256 residues in PDB (PDB 256). Left: box plot of RMSD between the generated structures and ESMFold predictions for the argmax sequence prediction for models trained on the synthetic dataset and PDB. The centre line indicates the median scRMSD, box boundaries indicate the 1st and 3rd quartiles, and whiskers show the 1st or 3rd quartile + 1.5 times the interquartile range from the box. Right: designability of argmax sequence predictions for models trained on synthetic data and PDB based on *n* = 200 generated backbones per condition.

As our models can be conditioned to generate proteins with a given secondary structure, we investigated if conditioning models with random secondary structures could increase the diversity of generated backbones. We sampled random three-state secondary structure strings (helix, strand and loop) by selecting a random percentage of helices and strands, constructing secondary structure elements of random lengths that add up to the selected percentages and randomly arranging them into a secondary structure string (Fig. 4a). We then used our denoising models to produce backbones for each random secondary structure. Computing diversity_designable_ for backbones of length 50–400 residues generated this way resulted in our models surpassing RFdiffusion at all sizes and matching or outperforming the designability of Genie 2 in spite of having been trained on a much smaller dataset^2^ (Fig. 4b). In particular, random secondary structure conditioning resulted in an increased diversity for small proteins and saturated the diversity metric on proteins of length 200 or larger. However, increasing diversity this way resulted in decreased designability across all protein lengths and models (Extended Data Fig. 5c).

In addition to greatly increasing clustering-based diversity, this approach equalized secondary structure content biases inherent to our models (Fig. 4c and Extended Data Fig. 5a,b,d). Although all of our models showed a preference for alpha-helices for unconditional generation, conditioning resulted in a uniform distribution of secondary structure content. Quantifying the diversity in secondary structure content of the designs showed that conditioning increased the entropy of the secondary structure distribution relative to the non-conditioned baseline (Extended Data Fig. 5e,f). This indicates that conditioned designs are more diverse both in terms of shape and secondary structure content.

To test the limits of random secondary structure conditioning for generating diverse protein structures, we generated a synthetic dataset of 50,000 backbones with size between 50 and 256 residues (Fig. 4d; also see the ‘Synthetic dataset generation’ section). We designed ten sequences per backbone with ProteinMPNN; predicted their structures with ESMFold; and quantified the designability, diversity and novelty with respect to the PDB. Of the 50,000 backbones, 81.4% were designable. Across protein sizes, designs showed low median scRMSD and high overall designability (Fig. 4e). To quantify diversity, we clustered all backbones using Foldseek with TM-align alignment (TM-score threshold of 0.6) and a minimum coverage of 90% of the sequence to only cluster structures of similar sizes^55^. This yielded 45,713 clusters corresponding to 91.4% of the dataset. Of these cluster representatives, 75.3% were designable, resulting in a dataset of 37,661 diverse and designable structures. Using Foldseek to search the PDB for matches for all designable structures in the dataset resulted in 11,973 structures without a single match at TM-score > 0.5. In particular, most matches were concentrated in short backbones, with the majority of backbones with 200 or more residues had no matches in the PDB. This indicates that generating structures with a random secondary structure can explore parts of the protein fold space far outside the training set and result in ‘dark matter’ folds outside the PDB.

### Synthetic data improve one-shot designability

Previous work on protein generative models^49,56^ reported that training on synthetic data with ProteinMPNN-designed sequence could improve model performance. To check if a synthetic dataset generated this way could be used to potentially train improved protein generative models, we compared the performance of two salad models trained on proteins of size 50–256 residues. We trained one model on a subset of PDB with chains of length between 50 and 256. The other was trained on designable structures and sequences in our synthetic dataset. Using each model, we generated 200 backbones for protein sizes between 50 and 300 residues. As our models learn to predict a sequence as an auxiliary task during training (see the ‘Denoising model loss’ section), we generated a single sequence per backbone. We predicted the structure of each sequence using ESMFold^24^ to assess design success. The model trained on PDB resulted in high median scRMSD (>2 Å) and low designability (<20%) across all tested protein sizes (Fig. 4f). By contrast, the model trained on our synthetic dataset showed low median scRMSD and high designability for in-distribution tasks, with performance deteriorating for 300-residue proteins, which the model was not trained on (Fig. 4f). Directly generating successful backbone–sequence pairs circumvents the sequence design step in the protein design pipeline, reducing the number of tested sequences and AlphaFold or ESMFold evaluations for design filtering from 8 to 1. This greatly decreases the runtime of the protein design pipeline.

### Structure editing for motif scaffolding

Although unconditional backbone generation can give an indication about the general performance of a protein generative model, it is rather removed from the realistic applications of protein generative models. Motif scaffolding provides a more realistic benchmark task. Models have to generate backbones that accommodate one or more functional motifs from natural proteins^1^. This has immediate applications in enzyme design (scaffolding theozymes)^15,16^, synthetic vaccine design^7^ and design of natural protein mimics^57^.

We compare the performance of salad models against the state-of-the-art protein diffusion models Genie 2 and RFdiffusion on a standardized motif-scaffolding benchmark. The benchmark, introduced in ref. 1, includes 24 single-motif tasks of varying difficulties and was extended in ref. 2 to contain six additional tasks in which the models have to scaffold more than one motif in a single backbone (multi-motif scaffolding). For a direct comparison with Genie 2 and RFdiffusion (which are both VP models), we only use VP models in this benchmark. As our models are not trained for multi-motif scaffolding by default, we approach this problem in two different ways. First, we use our structure-editing approach to edit the denoised structure by aligning the motif backbone and replacing the output coordinates by the motif’s coordinates (Extended Data Fig. 7a). This ensures that the motif is present in the final generated backbone, even if the model is not conditioned on the motif’s structure. We call this configuration salad+edit. Second, we train a separate multi-motif-conditioned model, which we will refer to as salad+cond (Extended Data Fig. 7b).

In the following we compare the results for our method with the results for RFdiffusion and Genie 2 reported in ref. 2. To directly compare with these, we closely followed the same evaluation strategy.

For each approach, we generated 1,000 backbones per scaffolding problem, designed eight sequences with ProteinMPNN and assessed the designability with ESMFold. We further filtered designable structures by their motif RMSD computed over all backbone atoms (N, CA, C and O). Structures were deemed successful if they reached motif RMSD < 1 Å. All successful structures were then clustered using TM-align at a TM-score cut-off of 0.6 to identify unique scaffolds for each problem. Evaluating success using CA-based motif RMSD showed little to no impact on both number of successful and unique designs for salad+cond (Extended Data Fig. 8a,b). By contrast, salad+edit showed large variability in success rates for some motifs (Extended Data Fig. 8a,b). This indicates that the lack of explicit motif conditioning may result in the model changing the motif orientation in the denoising step.

We found that both salad+edit and salad+cond solved 23/24 single-motif as well as 5/6 multi-motif design tasks (Extended Data Fig. 7c,d). Both salad+edit and salad+cond generated diverse backbones with low motif RMSD (Extended Data Fig. 7e). Only the motifs for 4jhw and 3ntn remained non-designable, consistent with Genie 2 (ref. 2). However, compared with Genie 2, we were able to solve one additional multi-motif-scaffolding task with 2b5i. Although RFdiffusion cannot be straightforwardly applied to multi-motif scaffolding, our approaches still outperformed it on single-motif scaffolding, solving one additional problem. Overall, salad+cond generated 1,610 (salad+edit, 1,446) unique scaffolds, slightly outperforming Genie 2 and dwarfing RFdiffusion’s 889 scaffolds (Extended Data Fig. 7c).

Thus, our models outperform RFdiffusion across all criteria, match Genie 2 in terms of the total number of unique scaffolds and solve one additional problem with 2b5i. Although a direct comparison of the number of unique backbones per scaffolding problem (Extended Data Fig. 8c) shows that there is currently no best model across all tasks, our models result in equal or more scaffolds for the majority of design tasks (21/24 versus RFdiffusion and 19/30 versus Genie 2 for structure editing; 20/24 and 20/30 for conditioning; Extended Data Fig. 8c). This indicates that both our approaches are competitive with the state of the art for single- and multi-motif scaffolding.

### Structure editing for repeat protein design

**Fig. 5:**
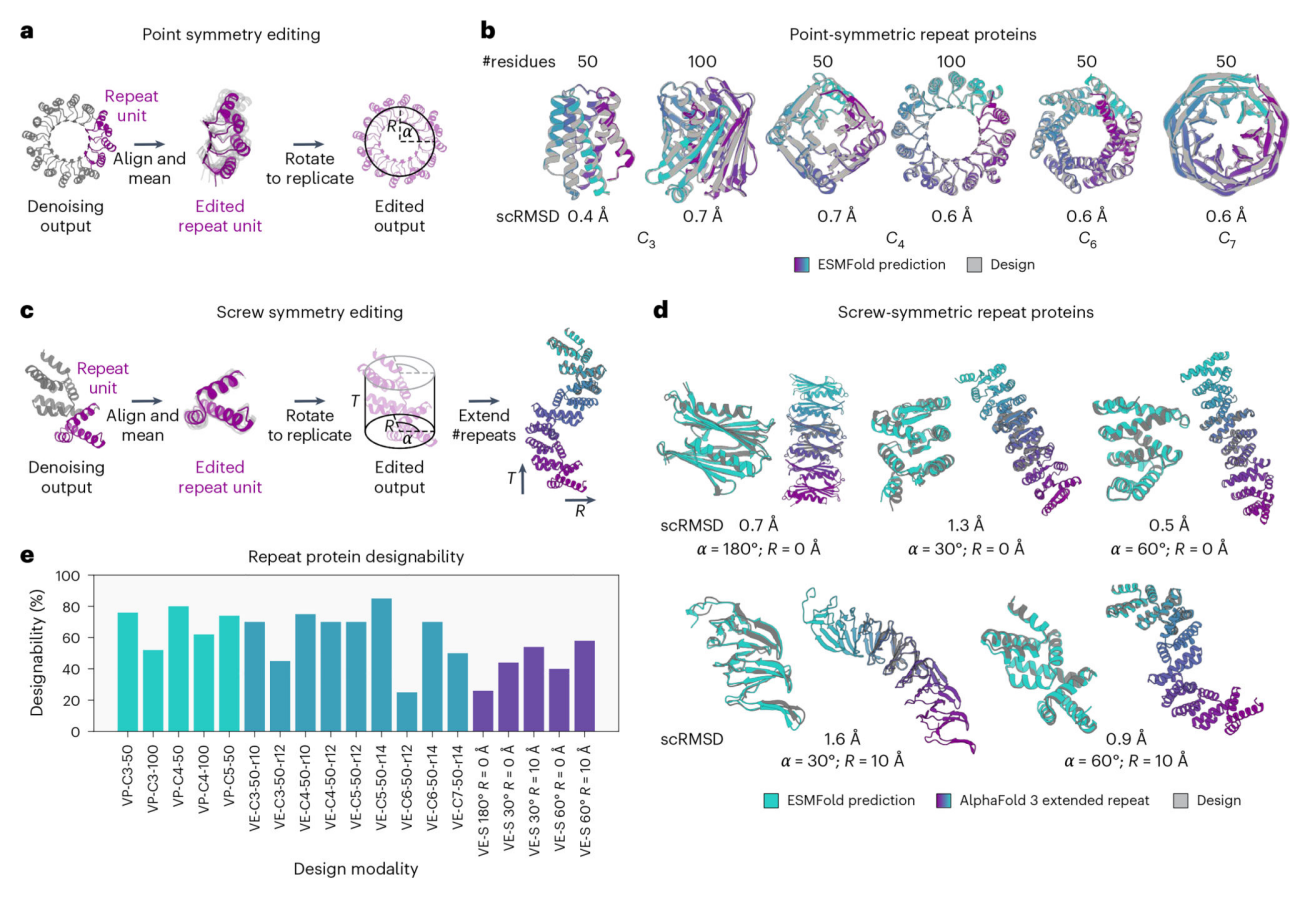
Edited denoising for symmetric and repeat proteins. **a**, Schematic of the structure editing procedure for point-symmetric repeat protein design. **b**, Example generated point-symmetric repeat proteins for different subunit sizes (50 and 100 residues) and cyclic groups (*C*_3_, *C*_4_, *C*_6_ and *C*_7_). The idealized symmetric design is shown in grey, and the ESMFold prediction for each design is coloured by the residue index. scRMSD values are reported for each example structure. **c**, Schematic of the structure editing procedure for screw-symmetric repeat proteins. **d**, Example screw-symmetric designs (grey) for different angles (30°–180°) and radii (0 Å and 10 Å), with a fixed inter-subunit translation of 12 Å. An ESMFold-predicted structure (turquoise) is superimposed onto the designed repeat (left). Additionally, a structure of the design predicted by AlphaFold 3 (ref. 72) replicated three times (coloured by residue index) is shown together with the scRMSD of the design to that extended repeat protein. **e**, Design success rates for cyclic and screw-symmetric designs using VP and VE models with different symmetry groups, radii and rotation angles based on *n* = 20 generated backbones per condition.

As a second application to demonstrate the flexibility of our models combined with structure editing, we set out to generate repeat proteins. Similar to the approach used in previous work^1^, we can generate point-symmetric repeat proteins by symmetrizing the inputs of our models according to the action of a point group (Fig. 5a). As our model has residues attend to random neighbours, we also symmetrize the output of our models. All repeat subunits are aligned using the action of the point-symmetry group and averaged to produce a representative repeat unit. This can then be placed at a specified radius from the symmetry axis to control the radius of the generated symmetric repeats. Replicating the representative structure then gives us a symmetrized output structure. Applying this procedure resulted in designable backbones with low scRMSD for various cyclic symmetry groups (Fig. 5b).

Although both hallucination^5^ and diffusion^1^ methods have been successful in producing point-symmetric proteins, only sequence-based^13^ machine learning methods and Rosetta-based protein design^3,4^ have been successfully applied to design extended repeat proteins that cannot be described by a point group. However, our editing approach is readily extended to arbitrary symmetry groups including screw (helical) symmetries, which cover the class extended repeats described in refs. 3,4,13 (Fig. 5c). By explicitly setting the radius *R*, rotation angle *α* and translation *T* of a screw-symmetric repeat (Fig. 5c), we can generate designable backbones with the specified geometry (Fig. 5d). Generated backbones are structurally diverse, with topologies ranging from fully alpha-helical to fully beta-sheet. Repeat sequences designed using ProteinMPNN^20^ are reliably predicted to take on the designed structure with low scRMSD, even when extending the number of repeats by a factor of 3 (Fig. 5).

Overall success rates for symmetric protein design vary between models (VP or VE) and design tasks (Fig. 5e). Strikingly, designabilities for both cyclic and screw-symmetric designs show a dependency on the specified radius and rotation angle, for example, *C*_6_ symmetry with a radius of 12 Å (r12) and a radius of 14 Å (r14). This is probably because the generated structures become highly compact for low radii, which is associated with a loss in designability (Extended Data Fig. 4).

### Structure editing for multi-state design

Although both motif scaffolding and repeat protein generation demonstrate the applicability of structure editing to protein design, these tasks do not showcase the full flexibility of this approach. In both cases, external conditioning information is available in the form of a motif or symmetry group. Additionally, there is sufficient data to train structure generative models conditioned on either of these tasks. By contrast, designing amino acid sequences that can fold into multiple distinct backbone structures (multi-state design) fits none of these criteria: data on natural multi-state proteins are scarce^58^ and structure-based generative models are believed to be unsuitable for this task^13^. Therefore, we chose to demonstrate that salad models can solve a recent multi-state benchmark task introduced in ref. 13 by using structure editing to couple the outcome of multiple denoising processes—one for each state.

**Fig. 6:**
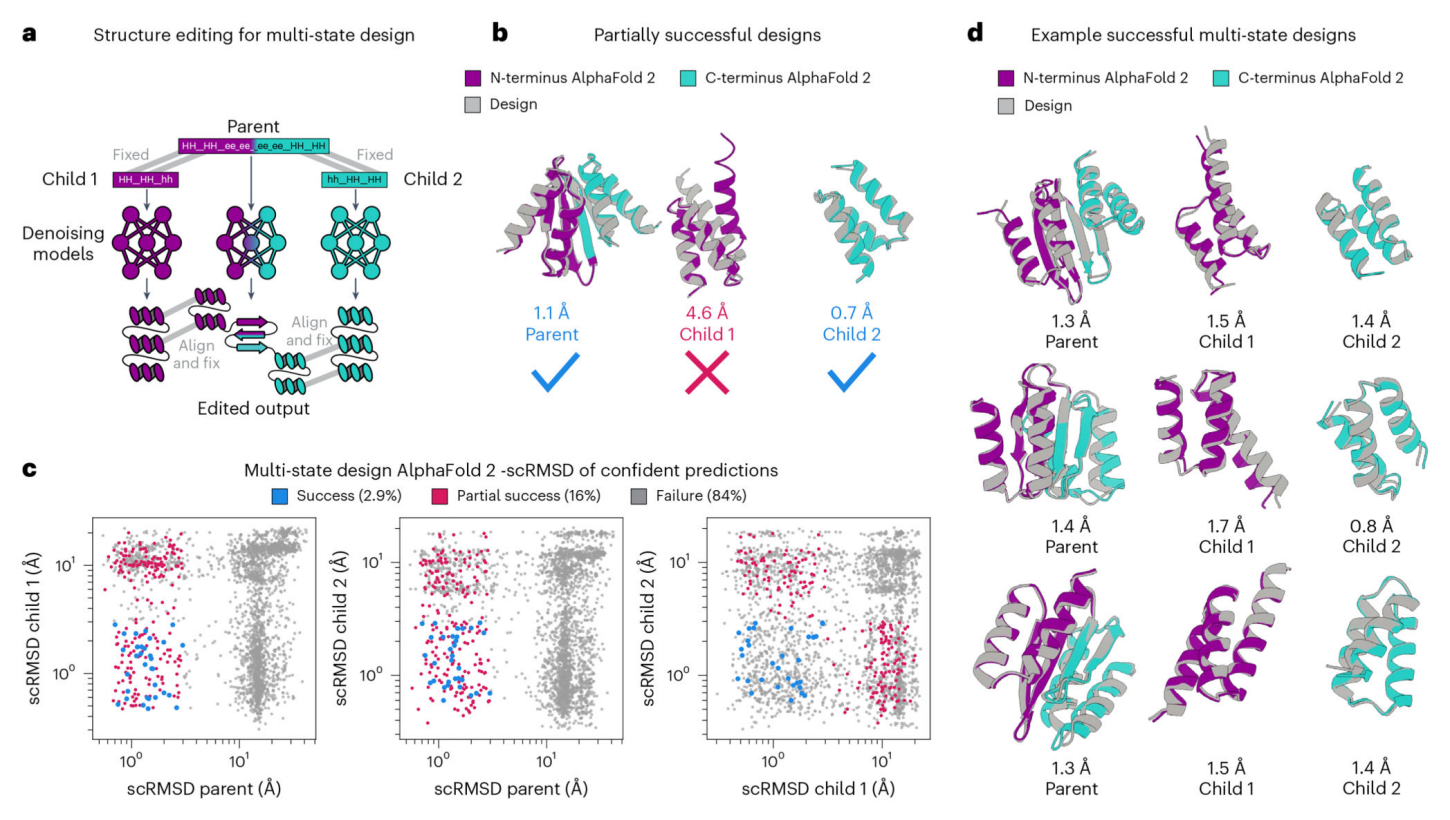
Multi-state protein design. **a**, Schematic of the structure editing procedure for multi-state protein design. **b**, Example partially successful multi-state design. **c**, Scatter plots of AlphaFold 2 scRMSD values for parent and child sequences with pLDDT > 75. Failed sequences are marked in grey, partial successes (parent and at least one child successful) are marked in purple and successful designs are marked in blue. **d**, Examples of successful multi-state designs. Generated structures for parent and child proteins are shown in grey; the predicted structures are coloured by their corresponding child protein. The N-terminal part is coloured purple and the C-terminal part is blue. AlphaFold 2 scRMSD values are reported for each parent and child design based on *n* = 1,000 backbones per condition.

Following ref. 13, we designed backbones for a protein (the ‘parent’) that takes on a specified secondary structure when intact and a different secondary structure when split into two ‘child’ proteins. The N- and C-terminal parts of the parent share their secondary structure with the children, whereas the central part of the parent should transition from a beta-sheet to an alpha-helix when split (Supplementary Table III). As we cannot directly design the protein on the sequence level, we instead instantiate three separate denoising processes, one for each state (Fig. 6a). Each denoising process is conditioned on the secondary structure string of either parent, child 1 or child 2. At each denoising step, we enforce that the parts with the same secondary structure between parent and child share a similar three-dimensional geometry by aligning and averaging their substructures (Fig. 6a). Essentially, the denoising processes are coupled by conditioning their structures on each other. This approach results in three coupled structures per generation.

We generated 1,000 structure triples, designed their sequences with ProteinMPNN, tying the sequence across parent and child structures^20^. To compare with the benchmark done in ref. 13, we evaluated the design success with the same criteria of AlphaFold RMSD < 3.0 Å and pLDDT > 75. Here 16% of the generated backbones resulted in partially successful designs, where all structures passed the pLDDT threshold, and a parent and at least one child passed the RMSD threshold (Fig. 6b). Although these designs did not meet all the criteria, they still resulted in a sequence predicted to change conformation in the parent state compared with the child state. Using the complete criteria, 2.9% of the backbones generated by our approach were successful, compared with the 0.05% of successes reported for ProteinGenerator (PG) in ref. 13. Although we used ProteinMPNN for sequence design, PG returns one sequence per backbone. However, even evaluating the percentage of success on a sequence-level salad reached a success rate of 0.32%, outperforming PG by a factor of 6.

To check whether structure editing contributed to the success rate of multi-state design, we generated another 1,000 backbone triples using only secondary structure conditioning. This resulted in per-backbone success rate of 0.4% and a per-sequence success rate of 0.06%, matching PG (Extended Data Fig. 9). This indicates that structure editing is indeed behind the increased performance observed for this multi-state design task. Overall, our structure editing approach generates varied low-RMSD solutions to this multi-state design problem (Fig. 6d), outperforming previous machine learning approaches. To our best knowledge, this is the first demonstration of multi-state design with a protein backbone denoising model.

## Discussion

In this work, we present salad, a family of efficient sparse denoising models, capable of generating designable and diverse protein structures up to a length of 1,000 amino acid residues. For unconditional protein structure generation, our models outperform RFdiffusion both in terms of designability and diversity for all protein lengths, closely approaching the diversity of Genie 2 (which was trained on a larger and more diverse dataset). We bridge this gap in diversity by applying random secondary structure conditioning at the cost of designability. This allows us to generate a large, highly diverse dataset of designable structures novel to the PDB. For longer proteins with 400–1,000 residues, our models clearly outperform both RFdiffusion and Genie 2, approaching the designability observed for hallucination-based methods^27^. Although the concurrently developed Proteina^42^ matches salad’s designability for up to 800-residue proteins, it does so at the cost of increased runtime, a 25-fold increase in the number of parameters, and does not generalize to 1,000-residue proteins. Therefore, salad pushes the boundaries of designability and diversity across protein lengths up to 1,000 and greatly reduces the time-per-design value due to its efficient sparse architecture. The ability to design large proteins in a high-throughput manner can open up new possibilities for designing increasingly large and complex molecular machines.

We expand the capabilities of salad by combining it with structure editing. By editing the output of a salad model at each denoising step, we can rapidly prototype generators for protein design tasks unseen during training. We show that this combination can generate designable, low-RMSD backbones for a variety of tasks. We design shape-conditioned proteins, scaffold multiple functional motifs, generate repeat proteins and produce protein sequences predicted to adopt distinct folds when cleaved. For motif scaffolding, salad matches or exceeds the performance of both RFdiffusion and Genie 2. For repeat protein design, we generate screw-symmetric repeats, which, to our knowledge, have not been explored using protein structure diffusion models beyond a single mention in ref. 21. Instead, such proteins have so far been designed using Rosetta^4^ or sequence-based design methods^3,13^. For multi-state protein design, we reproduced a design task introduced in ref. 13 and achieved a success rate one order of magnitude higher than the original work. This indicates that salad is, in fact, sufficiently flexible to generate designs even for tasks like multi-state design, which are believed to be unfavourable for structure generative models^13^.

Although salad produces acceptable results on computational benchmarks, this work does not contain additional experimental validation. While our ESMFold- and AlphaFold-based approach to measure designability have been previously shown to select experimentally viable protein designs^1,13,20,28^, it is ultimately not the ideal metric. Neither AlphaFold nor ESMFold can perfectly distinguish experimentally viable from non-viable designs^28,59^—both models are known to be vulnerable to adversarial protein sequences^5,27,32^ and have limited sensitivity to amino acid masking and mutation^59^. Nonetheless, prior work has produced extensive experimental validation, showing that a pipeline using a structure generator with ProteinMPNN sequence design and structure predictor filtering can produce experimentally viable designs at a reasonable rate^1,13,20^. We argue that salad matching or exceeding previous, experimentally validated models in terms of ESMFold/AlphaFold design success alleviates concerns about the lack of experimental validation. In particular, salad is part of the same pipeline of structure generation, ProteinMPNN sequence design and AlphaFold 2/ESMFold selection as RFdiffusion^1^. As salad was trained independently from ProteinMPNN and AlphaFold 2/ESMFold, it is unlikely that it has learned to generate backbones that are adversarial for both. We also emphasize that the focus of this work is on developing more efficient and versatile backbone generators, not to present an all-in-one solution for protein design.

Another limitation of salad is that it is currently restricted to a limited training set. salad is trained on protein structures in the PDB, with all small molecules, ions, waters and nucleic acids removed. Therefore, its uses for enzyme design and small-molecule binder design are limited. In particular, more recent versions of RFdiffusion can design proteins in the presence of small molecules^16,19^. The salad architecture is likely capable of handling small molecules with minor modifications, which makes this an attractive step to address in the future. In addition, salad struggles to match the diversity of Genie 2 (ref. 2) without using random secondary structure conditioning. Genie 2 was trained on a clustered subset of the AlphaFold database, which greatly exceeds our PDB dataset in both size and structural diversity^2,54,60^. We believe that this issue can be addressed in future work by training salad models on AlphaFold DB.

In this work, we compare salad to RFdiffusion^1^ and Genie 2 (ref. 2) as well as the concurrent work of Proteina^42^. Although many other protein diffusion models exist^37,40,61–63^, we argue that comparing to these ones in particular is sufficient to establish salad to be on par with the state of the art, as prior work has shown that they outperform most other protein structure generative models^1,2,42^. The designability of salad generations for up to 1,000-residue-long proteins as well as its runtime performance give it an edge over comparable models. This should enable salad to fill the niche it is designed for, providing efficient and versatile backbone generation in the first step of the protein design pipeline.

## Methods

### Protein structure denoising models

Denoising protein structure models are trained to reconstruct a noise-free structure **x** from a noisy input structure. To train our models, we sample random time points *t* ∈ [0, 1], where *t* = 0 corresponds to a noise-free structure and *t* = 1 corresponds to pure noise. Depending on the noise schedule, we then convert *t* into a noise scale *σ*_*t*_. We use three different noise schedules: VP noise with a cosine schedule^64^ and constant-standard-deviation noise (VP), VP noise with a protein-size-dependent standard deviation (VP scaled) and VE noise (VE).

For VP noise, a noisy structure **x**_*t*_ is then generated by sampling

(3) $$x_{t}^{\text{VP}}(x)\sim N\left(\sqrt{1-\sigma_{t}^{2}}\cdot x,\sigma_{t}^{2}\cdot \sigma_{\text{noise}}^{2}\right),$$

where *σ*_noise_ = 10 Å is the standard deviation of the noise at time *t* = 1. For VP-scaled noise, we instead set the standard deviation *σ*_noise_ equal to the standard deviation of CA positions in input structure **x**:

(4) $$X_{t}^{\text{VP}\;\text{scaled}}(x)\sim N(\sqrt{1-\sigma_{t}^{2}\cdot x,\sigma_{t}^{2}\cdot \sigma {\left(x_{\text{CA}}\right)}^{2}),}$$

The fully noised structure $x_{1}^{VP-\;scaled}$ will, therefore, have the same standard deviation as the alpha-carbons starting structure **x**_CA_. Finally, for VE noise, instead of sampling diffusion time *t*, we directly sample a noise scale from a log-normal distribution, following ref. 65:

(5) σ~LogNormal(1.6,1.4)Å

and sample noisy structures according to

(6) $$x_{\sigma }^{\text{VE}}\sim N\left(x,\sigma^{2}\right).$$

The models are then trained to reconstruct **x** from **x**_*t*_ by minimizing $∥x-x_{t}{∥}_{2}^{2}$ and additional auxiliary losses (see the ‘Denoising model loss’ section).

Our models are trained using self-conditioning. At each training step, we sample two noisy structures **x**_*t*_ and $x_{t}^{\prime }$. With probability 50%, we predict **x**′ from $x_{t}^{\prime }$ without self-conditioning. We then predict **x** from **x**_*t*_ using **x**′ as an additional input. At inference, the model passes the current noised structure **x**_*t*_ as well as its previous prediction **x**_prev_ to predict **x**.

In addition to self-conditioning, our models can also be conditioned on amino acid sequence, partial structure information, three-state secondary structure (helix, strand and loop), block contacts between secondary structure elements, inter-chain contact information and hotspot residues interacting with other protein chains^1^. During training, conditioning information is provided at random for 50% of training examples. Each conditioning modality is further randomly masked for a random fraction between 20% and 100% of the residues. To determine inter-residue and inter-chain contacts for conditioning, we compute pairwise CA distances between residues. Residues are considered in contact, if their CA distance is < 8 Å. Two chains and secondary structure elements are considered in contact if at least one pair of residues is in contact. Residues are considered hotspot residues if they are in contact with at least one residue in another chain. Partial structure information is presented to the model as a matrix of inter-residue CB distances together with a mask of amino acid pairs with valid conditioning information. Partial structure information is masked out for between 20% and 80% of residues in any training example.

To sample from a trained denoising model, we initialize a backbone **x** with all atom positions set to 0. We then partition the interval [0, 1] into *N* equally spaced time steps (*t*_0_…*t*_*N*_) with *t*_*N*_ = 1. For each time step *t* starting with *t*_*N*_, we apply noise with a chosen noise schedule to **x**, resulting in a noised structure **x**_*t*_. This noisy structure is denoised by the model, resulting in a new structure **x**. Repeating this process gradually reduces the noise level and results in a denoised protein backbone. Our approach differs from the denoising processes described in the literature: protein structure denoising diffusion models generally sample structures **x**_*t*_ according to a distribution *q*(**x**_*t*_|**x**_*t*+*s*_, **x**), which depends on both denoised structure **x** and an earlier noisy structure **x**_*t*+*s*_ at diffusion time *t* + *s* (refs. 1,2,35). Instead, our approach samples from *q*(**x**_*t*_|**x** = *f*_*θ*_(**x**_*t*+*s*_)), removing any direct dependency on the previous noise **x**_*t*_ and only depends on it through the model *f*_*θ*_. This approach has been previously reported for categorical text diffusion models^66^ and more recently for amino acid sequence diffusion models^13^.

We chose this approach not because of its success in sequence diffusion models but to enable the arbitrary modification of denoised structures **x** without having to take into account **x**_*t*_. To use our models for protein generation tasks they were not trained for, we wanted to allow the arbitrary editing of the denoised structure—for symmetrization, to introduce structural motifs for scaffolding and to couple multiple denoising processes for multi-state design. This necessitates translating, rotating and replacing parts of the denoised structure. Changing the denoised structure **x** this way without also adjusting **x**_*t*+*s*_ in a compatible way could result in failure to generate valid protein structures.

Supplementary Algorithm 1 shows the generative process for a model involving conditioning information *c*, self-conditioning and structure editing.

### Model architecture

Our sparse denoising models consist of three separate modules. An Encoder that encodes the ground-truth backbone atom positions (N, CA, C, O and idealized CB) **x**_gt_ and adds 15 additional pseudoatom positions for each residue to result in the denoising model input **x**, a DenoisingModule that receives noised positions **x**_*t*_, and is trained to reconstruct **x** and an amino acid decoder (AADecoder), which predicts an amino acid sequence and side-chain conformations for each residue. The model is trained with self-conditioning, receiving a previously predicted structure **x**_prev_ and per-residue representation local_prev_ as additional inputs. All modules are based on a sparse transformer architecture^44^ with pre-normalization^45^.

#### DenoisingModule

The DenoisingModule consists of six denoising blocks based on a pre-norm transformer architecture^45^. Every block updates the per-residue representation local_*i*_ of size local_size = 256 and residue atom positions **x**. We save the trajectory of **x** values across all blocks to apply losses over the entire denoising trajectory.

Supplementary Algorithm 2 shows an overview of a block in the DenoisingModule. We replace standard self-attention in the transformer block by a sparse version of IPA (SparseIPA)^23^. Instead of computing the attention matrix and pair features for all amino acid pairs, we compute them for a set of precomputed neighbours. This reduces the complexity of attention from *O*(*N*^2^) to *O*(*N* · *K*), where *K* is the number of neighbours per residue. To support conditioning on structure information, we use two SparseIPA layers. The first IPA layer operates on the current set of position features, whereas the second one operates on previous positions from self-conditioning, as well as block contact and distance conditioning information. For multi-motif models, we instead run IPA using motif information first, followed by IPA on the current position features.

Following SparseIPA, the per residue features local_*i*_ are updated using a GeGLU-gated feed-forward layer with global pooling of the hidden state (Update; Supplementary Algorithm 5). This combination of sparse attention and global mean pooling of features allows the DenoisingModule to learn global dependencies without having to use full *O*(*N*^2^) attention.

#### Neighbour selection

To compute sparse attention features, we select a set of neighbours for each residue based on their sequence and CA distances. For each residue, we choose the 16 nearest neighbours by residue index. Then, we select an additional 16 neighbours by CA distance, excluding previously selected neighbours. Finally, we select #random neighbours at random with probability $1/d_{CA}^{3}$ following ref. 21 and #cond neighbours based on pairwise conditioning information, such as block contact conditioning^1^ or pairwise distances. All default models have #random = 32 when computing neighbours on the current set of positions and #random = #cond = 16 when computing neighbours on self-conditioning information. This results in a total of 64 neighbours per amino acid. Multi-motif-conditioned models have #random = 32 and do not use additional neighbours from the conditioning information. It is important to note that unlike Chroma^21^, a new set of neighbours is computed for each DenoisingBlock, as each block updates the residue positions **x**.

#### Pair features

As part of SparseIPA, we compute amino acid pair features for each amino acid and its selected neighbours. Distances between all pairs of backbone atoms (N, CA, C, O and idealized CB) are computed for each pair and featurized using 16 Gaussian Radial Basis Functions^20^ uniformly spaced between 0 Å and 22 Å. The bandwidth is set to the distance between Radial Basis Function centres *σ* = 1.375 Å. In addition to distance features, we also compute direction from the CA atom of each residue to each of its neighbour, the relative rotation between residues and the atom positions of a residue and its neighbour in local coordinates. These features are then flattened and linearly projected to pair_size = 64 pair features. For models with minimal pair features, we instead only compute inter-residue distances and relative rotations (Supplementary Algorithm 4).

#### Update module

After applying SparseIPA, we update per-residue features local_*i*_ using a gated feed-forward layer. We first update local_*i*_ using the atom positions in each residue. Then, we linearly project and pool amino acid features within and across chains. Per-residue, per-chain and per-complex features are then summed and passed through a final linear layer (Supplementary Algorithm 5). Combined with SparseIPA, this allows the model to learn global dependencies within a protein complex without the need for full *O*(*N*^2^) attention.

#### Equivariant position update

The final component of a Denoising-Block updates the atom positions **x**_*i*_ for each residue in an equivariant manner. Per-residue features local_*i*_ are linearly projected to a set of position updates, scaled by a unit factor of 10 Å and added to the current positions **x**_*i*_ in the local frame of each residue *i*. The resulting updated positions are then transformed back into global coordinates (Supplementary Algorithm 6).

#### Structure encoder

The Encoder uses a simplified version of the DenoisingModule and uses the same feature size as the main DenoisingModule. As the Encoder does not change the protein backbone **x**_gt_, we use a precomputed set of neighbours for each residue. Each amino acid is assigned a set of 32 nearest neighbours based on the CA distance. The trunk of the Encoder consists of two blocks of SparseIPA followed by a GeGLU layer^46^. After the second block of the Encoder, the residue representation is used to generate 15 pseudoatom positions per residue, which are combined with the backbone atom positions. The resulting structure **x** is used to train the DenoisingModule.

#### AADecoder

The AADecoder uses three blocks of the same type as the Encoder, together with a set of 32 nearest neighbours per amino acid computed on the denoised CA positions. In addition to denoised positions **x** and DenoisingModule features local_*i*_, the AADecoder also receives a partially masked amino acid sequence during training. A random fraction between 1% and 100% of amino acids in each training sequence are replaced by a mask token. The AADecoder is then trained to predict the masked amino acids with a cross-entropy loss. This corresponds to the training objective of an autoregressive diffusion model^67^.

#### Model variants

We trained denoising models for three different noise schedules: VP with *σ* = 10 Å; VP with *σ* = *σ*(**x**_CA_) dependent on the standard deviation of CA atoms in the training example; and VE diffusion with *σ* ~ LogNormal (1.4 Å, 1.6 Å). For each noise schedule, we trained three ablated models: a model with full pair features and Fourier time embedding^35^; a model without time-embedding features; a model with minimal pair features and no time-embedding features.

### Denoising model loss

Our denoising models are trained using a combination of standard denoising and auxiliary losses. A per-block denoising loss is computed on residue (pseudo) atom positions for the output $f_{b}^{ar}\left(x_{t}\right)$ of each DenoisingBlock, where *r* are residues and *a* are the atoms in each residue:

(7) $${ℒ}_{b}\left(x,x_{t}\right)=\frac{1}{N_{a}\cdot N_{r}}\underset{a,r}{\sum }\text{clip}{\left(∥f_{b}^{ar}\left(x_{t}\right)-x{∥}_{2},0,10\;\text{Å}\right)}^{2}.$$

The norm ||**x**_*b*_ − **x**|| is clipped to 10 Å to stabilize training and the loss is averaged over residues *r* and (pseudo) atoms *a* in each residue. The losses for each block are then weighted together to result in a trajectory denoising loss:

(8) $${ℒ}_{\text{traj}}=2\cdot {ℒ}_{n}\left(x_{t},x\right)+\frac{1}{n}\sum_{b=1}^{n}{ℒ}_{b}\left(x_{t},x\right)$$

where the final prediction is weighted by a factor of 2 to increase its importance in the final loss. This is combined with an auxiliary all-atom denoising loss using the all-atom structure $f_{atom\;}^{ar\;}\left(x_{t}\right)$ predicted by the AADecoder:

(9) $${ℒ}_{\text{atom}}=\frac{1}{N_{r}}\underset{r}{\sum }\frac{1}{N_{a}}\underset{a\in r}{\sum }\text{clip}{\left(∥f^{ar}\left(x_{t}\right)-x_{\text{gt}}{∥}_{2},0,10\;\text{Å}\right)}^{2}.$$

To ensure that the models learn to reproduce the relative orientations between amino acid residues, we also introduce a rotation denoising loss for each block following RFdiffusion^1^:

(10) $${ℒ}_{\text{rot},b}=\frac{1}{N_{r}}\underset{r}{\sum }∥R_{r}^{\text{T}}R_{r,\text{gt}}-1{∥}_{2}^{2},$$

where **R**_*r*_ and **R**_*r*,gt_ are the rotation matrices defined by the backbone frame of each residue in the predicted and ground-truth structures, respectively^1^. This results in a trajectory denoising loss for residue rotations as

(11) $${ℒ}_{\text{rot}}=2\cdot {ℒ}_{\text{rot},n}+\frac{1}{n}\sum_{b=1}^{n}{ℒ}_{\text{rot},b}.$$

In addition to using unaligned denoising losses, we also compute a squared frame-aligned point error (FAPE) loss ${ℒ}_{\text{FAPE}}^{2}$ (ref. 23) over the trajectory of predictions $f_{b}^{ar}\left(x_{t}\right)$ as well as a local FAPE loss on the predicted all-atom structure ℒ_*local*_. Instead of computing the FAPE over all amino acid pairs, we instead compute it over the 64 nearest neighbours in the ground-truth structure ℒ_*fape*_ and 16 nearest neighbours for ℒ_*local*_. As with the denoising loss, the FAPE losses are also clipped to a maximum of 10 Å. Finally, the structural losses also include AlphaFold’s structural violation loss ℒ_*viol*_ (ref. 23) to penalize clashes in denoised structures.

The models are also trained with a number of non-coordinate losses, consisting of a distogram ℒ_*dist*_ (ref. 23) and amino acid prediction ℒ_*aa*_ and secondary structure ℒ_*dssp*_ cross-entropy losses.

(12) $${ℒ}_{\text{aux}}=10\cdot {ℒ}_{\text{aa}}+{ℒ}_{\text{dssp}}+0.1\cdot {ℒ}_{\text{dist}}$$

The final weighted loss of the model is then as follows.

(13) $$ℒ={ℒ}_{traj\;}+{ℒ}_{\text{atom}}+{ℒ}_{\text{fape}}+{ℒ}_{\text{rot}}+10\cdot {ℒ}_{\text{local}}+10\cdot {ℒ}_{\text{aa}}+0.1\cdot {ℒ}_{\text{viol}}+{ℒ}_{\text{aux}}$$

In this loss, ℒ_*viol*_ and ℒ_*local*_ are set to zero in the high-noise regime (diffusion time *t* > 0.5 for VP models; noise *σ*_*t*_ > 5.0 Å for VE models), as the model is unlikely to learn to predict non-clashing structures at high noise levels.

### Structure autoencoder models

Our sparse autoencoders were implemented to have the same graph transformer architecture as the denoising models. Each autoencoder model consists of a single Encoder block with SparseIPA over 32 nearest neighbours for each residue, followed by a GeGLU layer^46^. The resulting per-residue representation local_*i*_ is then layer normalized^68^ and linearly projected to a latent vector *z*_*i*_ : ℝ^latent_size^ for each residue *i*. For model variants with VQ^48^ enabled, *z*_*i*_ is then quantized with a codebook of size 4,096.

This latent representation is decoded by a Decoder, which consists of six blocks of SparseIPA followed by the same Update and position_update layers used in our denoising models. The decoder is trained with zero to three recycling steps for each batch, initializing the positions **x** of each recycling step with the result of the previous step **x**_prev_. The first recycling step starts from randomly initialized positions **x** ~ 𝒩(0, 1). We train models with three different decoder variants: an SE(3) equivariant model using the same neighbour selection and features as used in our denoising models; an equivariant model with per-block distogram prediction (EQ+dist; Supplementary Algorithm 7) and an additional SparseIPA layer using distogram nearest neighbours (Supplementary Algorithm 8); and a non-equivariant model directly embedding atom positions without first projecting them to residue local coordinate systems. This results in a total of six models trained (three decoder variants with and without VQ).

All the models are trained with ${ℒ}_{\text{FAPE}}^{2}$ over the entire denoising trajectory and ℒ_local_ for the final all-atom structure prediction. Amino acid cross-entropy ℒ_aa_ is used as an auxiliary loss. In addition, models with per-block distogram loss are trained with distogram cross-entropy for each layer ℒ_dist_. This results in a combined autoencoder loss of

(14) $$ℒ={ℒ}_{\text{FAPE}}^{2}+{ℒ}_{\text{local}}+{ℒ}_{\text{dist}}+10\cdot {ℒ}_{\text{aa}}.$$

### Training dataset

We trained our models on a snapshot of PDB collected in October 2023 excluding any PDB entries submitted after 31 December 2020. PDB entries were then filtered for a resolution of ≤ 4 Å. Entries containing protein chains of length less than 16 were excluded from training and non-amino-acid residues were removed from chains in the dataset. We clustered all protein chains in the resulting dataset using mmseqs2 (git commit 4f046dd)^69^ with a 30% sequence identity cut-off. To train our model, we generated input batches of 1,024 residues. Batches were constructed by repeatedly sampling structures from the dataset, until the total number of residues reached 1,024. If the total number of residues would exceed 1,024, the batch was zero padded instead, and the sampled structure was included in the next batch. At each epoch, we sampled clusters from the dataset without replacement, selecting a random chain identifier and biological assembly for each cluster. If the selected chain belonged to a complex and the entire complex fit in the current batch, we added the complex to the batch with a probability of 50%. Otherwise, we added only the selected chain.

### Model training

All the denoising models were trained for 200,000 iterations on the dataset with a mini-batch size of 1,024 and 32 batches per iteration, resulting in a total of 32,768 residues per iteration. Structure autoencoder models were trained for 200,000 steps with a batch size of 16,384 residues. We used the Adam optimizer^70^ with *β*_1_ = 0.9 and *β*_2_ = 0.99. The learning rate was warmed up from 0 for 1,000 steps at the start of training and then reduced to 1 × 10^−7^ using cosine decay^71^. On an example machine with eight NVIDIA RTX 3090 GPUs, an average training run took 3.5 days, or 672 GPU hours. Models were trained on different GPU nodes using eight of either NVIDIA RTX 3090, A40 or L40S GPUs.

### Runtime benchmarking

We compared the runtimes of salad models with RFdiffusion, Genie 2, Chroma and Proteina on a single NVIDIA RTX 3090 GPU. We sampled ten structures from each model using their default settings (Supplementary Table I) and measured the time elapsed for each generated structure. We discarded times measured for the first generated structure to account for library initialization and model compilation and reported the average time for the remaining nine generations.

### Model ablation study

We selected a model architecture and sampling hyperparameters by evaluating models with and without time-embedding features as well as with full and minimal pair features on unconditional structure generation. We generated 200 backbones for proteins of size 50–400 residues for each model, using 100, 200 and 500 diffusion steps with early stopping at 80, 180 and 400 steps into the denoising process. Self-conditioning was applied until diffusion time *t*_prev_ = 0.8 for VP models as this was determined to yield good results in preliminary testing. For VE models, we tested self-conditioning thresholds of 0.8 and 0.99. Ten sequences were designed for each backbone using ProteinMPNN^20^. Structures were predicted using ESMFold^24^. Designability was measured as the fraction of backbones with at least one designed sequence with pLDDT > 70 and scRMSD < 2.0 Å (Extended Data Fig. 6a,b). Models with full pair features, time embedding and 500-step sampling were chosen for further benchmarking. *t*_prev_ = 0.99 was chosen for VE models.

### Unconditional generation benchmark

At each evaluated protein length between 50 and 1,000 residues, we generated 200 protein backbones using both our models as well as Genie 2, RFdiffusion and Proteina for comparison^1,2,42^. Backbones were sampled using 500 diffusion time steps with early stopping at 400 time steps and self-conditioning turned off below the threshold diffusion time *t*_prev_ = 0.8 for VP models and *t*_prev_ = 0.99 for VE models. For each backbone, we then generated ten amino acid sequences using ProteinMPNN with a temperature of 0.1 (ref. 20). This resulted in a total of 11 sequences for our models (ten ProteinMPNN and one from the model itself) compared with ten sequences for Genie 2 and RFdiffusion. To fairly measure the model performance and remain comparable to previous work, we restricted all the computed performance measures to use the first eight sequences generated by ProteinMPNN.

We predicted the structures of each sequence using ESMFold^24^ and AlphaFold 2 (ref. 23). For each structure prediction, we measured the RMSD to the generated backbone (scRMSD) and pLDDT. Following ref. 2, we then computed designability as the fraction of the generated backbones with at least one sequence with ESMFold pLDDT > 70 and scRMSD < 2 Å. We evaluated pairwise similarities between the generated backbones using TM-align (v.20220412)^29^. To compute backbone diversity for direct comparison with Genie 2 and RFdiffusion^2^, we randomly subsampled the set of generated structures to a size of 100 backbones. Designable structures in this subset were then clustered using single-linkage clustering on the TM-score. Backbones with TM-score > 0.6 were included in the same cluster. Diversity for all backbones (diversity_all_) was then defined as the fraction of designable clusters in all generated structures $\frac{\;\#\text{clusters}\;}{\;\#\text{generated}\;}$ (ref. 2). We also defined a second diversity measure as the fraction of clusters in all designable structures $\;{\text{diversity}}_{designable\;}=\frac{\text{\#}\;\text{clusters}\;}{\text{\#}\;\text{designable}\;}$ to fully separate diversity from designability. Diversity was measured on ten samples of 100 structures, each sampled from the original 200 generated structures to report median, minimum and maximum diversities for each model.

### Shape-initialized structure generation

We prepared letter shapes as paths in SVG format using Inkscape 1.4 (e7c3feb100, 2024-10-09; Inkscape Project) and then extracted the coordinates of the nodes in each path into a CSV file. To sample structures based on these shapes, we used our VE model with default settings and shaped noise initialization. Instead of initializing the denoising process with noise for each residue *i* as

(15) $$\epsilon_{i}\sim N\left(0,{\left(80\;\text{Å}\right)}^{2}\right),$$

we instead centred the noise on the coordinates of nodes of the SVG path corresponding to the desired shape:

(16) $$\epsilon_{i}\sim N\left({\text{node}}_{i},{\left(80\;\text{Å}\right)}^{2}\right),$$

where node_*i*_ is the position of the node assigned to residue *i*. To generate the letter shapes described in this work, we assigned 200 consecutive residues to each node, that is, residues 1–200 were assigned to the first node, 201–400 to the second and so on. We then sampled ten structures for each letter shape (S, A, L and D) starting from this noise, designed ten sequences with ProteinMPNN at a temperature of 0.1 and predicted their structures with ESMFold. We then identified designable structures as described in the ‘Unconditional generation benchmark’ section.

### Shaped noise

To initialize noise for VE models better suited for large protein generation than normal-distributed noise, we adapted shape-initialized noise generation to work with random noise centres. We sampled random starting positions for centres centre_*i*_ according to

(17) $$\;{\text{centre}}_{i}=\underset{k<i}{\sum }{\text{centre}}_{k}+\epsilon_{i}$$

where *ϵ*_*i*_ ~ 𝒩 (0, (10 Å)^2^) is a normal-distributed offset. Essentially, we are sampling centres as Gaussian chains with average segment length of 10 Å (ref. 21). We then enforce globularity of the chain by optimizing inter-chain distances with a harmonic restraint centred on 10 Å. Optimization is done using ten steps of gradient descent with a learning rate of 0.1. We sample shape-initialized noise with 200 residues per centre using these randomly generated centres. We generate fresh centres for each designed backbone.

### Random secondary structure conditioning

To sample a random three-state secondary structure (helix, strand and loop) of a fixed length, we first sampled a random secondary structure fraction with a maximum loop content of 50% and arbitrary proportions of alpha-helices and beta-strands. We then computed the closest integer number of helix, strand and loop residues for this fraction at a fixed protein length. To arrange these residues into contiguous secondary structure elements, we then heuristically determine the minimum and maximum numbers of helices and strands that can be generated using this number of residues (Supplementary Table II). We sample a random number of helices and strands in this range and randomly assign residues to each helix and strand until we reach the previously computed number of residues for each secondary structure. These secondary structure elements are then randomly shuffled, and the remaining loop residues are randomly placed in between.

For random secondary structure sampling, we then conditioned our models on secondary structure strings generated this way, randomly replacing secondary structure elements with unknown secondary structure with a probability of 50% per element. Additionally, the first and last residues in each secondary structure element were replaced with an unknown secondary structure to allow the model to decide the correct secondary structure at boundaries between secondary structure elements. Evaluation of structures generated using random secondary structures followed the procedure described in the ‘Unconditional generation benchmark’ section.

### Synthetic dataset generation

To generate our synthetic protein dataset, we used our VP model with random secondary structure conditioning. We generated 50,000 backbones for random protein lengths between 50 and 256 residues. For each backbone, we designed ten sequences using ProteinMPNN with a temperature of 0.1 (ref. 20) and predicted their structures using ESMFold^24^. We then identified successfully designed sequences with scRMSD < 2 Å and pLDDT > 70. The dataset was then restricted to structures with at least one successful sequence, resulting in 41,713 backbones. These backbones were then clustered using Foldseek^55^ with a TM-score cut-off of 0.6 and minimum coverage of query and target of 0.9 using the command foldseek easy-cluster data/pdb/-c 0.9 –tmscore-threshold 0.6. The coverage cut-off was chosen in this way to mostly cluster structures of similar size. This resulted in 37,661 structures chosen as cluster representatives and also had one or more successful sequence designs, corresponding to 90.3% of designable backbones. We evaluated the percentage of novel structures relative to PDB by running Foldseek^55^ against PDB using TM-align and exhaustive search (foldseek easy-search data/pdb/fs_pdb –alignment-type 1 – format-output query,target,alntmscore,qtmscore,ttmscore,alnlen, qstart,qend,tstart,tend, where fs_pdb is a precomputed copy of the PDB database downloaded using Foldseek). Structures were considered novel if they had no match in the PDB with query TM-score > 0.5 (qtmscore).

### Synthetic dataset model benchmark

We trained two salad models with default_vp configuration on both the synthetic dataset and the PDB dataset described above, limited to sampling only single chains of length between 50 and 256 residues. Models were trained according to the procedure in the ‘Model training’ section. We assessed the performance of both models using the ESMFold structure prediction of a single sequence prediction for each generated backbone according to the procedure in the ‘Unconditional generation benchmark’ section. Instead of using ProteinMPNN^20^ for sequence design, we directly used the sequence defined by the argmax of the amino acid distribution predicted by each model at the final denoising step.

### Motif-conditioning model training

To compare with Genie 2 (ref. 2), we trained a separate salad model with multi-motif conditioning. The model was trained on PDB and was given multi-motif-conditioning information for each training example. Training was run for 200,000 steps according to the procedure described in the ‘Model training’ section. To prepare the motif-conditioning information, we first partitioned each structure into contiguous segments with random lengths between 10 and 50 residues. Each segment was then assigned to one of two segment groups. Only segments within the same segment group would then be treated as a single rigid segment for the purpose of multi-motif scaffolding. Finally, segments were set as active with a probability of 50%. Inactive segments were not used for conditioning. We then computed the CA distance map between all amino acids, together with a mask indicating amino acid pairs with active conditioning:

(18) $${\text{mask}}_{ij}=\left(s_{i}=s_{j}\right)\wedge a_{i}\wedge a_{j},$$

where *s*_*i*_ is the segment ID of a residue and *a*_*i*_ is a Boolean specifying if the segment at that residue is active.

### Motif conditioning using structure editing

In addition to training a model for multi-motif scaffolding, we adapted the sampling process of the default_vp model to allow multi-motif design. At each denoising step, we align the motifs to its corresponding residues in the denoised structure. We then replace the coordinates of those residues with the coordinates of the motif (Supplementary Algorithm 9). Sampling structures in this way guarantees that the motif will be incorporated into the resulting backbone.

### Motif benchmark

Following ref. 2, we generated 1,000 structures using motif conditioning and motif editing for each single-motif-scaffolding task defined in ref. 1 and additional multi-motif-scaffolding task specified in ref. 2. We then designed sequences for each backbone using ProteinMPNN and evaluated the designability using ESMFold (see the ‘Unconditional generation benchmark’ section). In addition, we computed the CA and full-backbone (N, CA, C and O) RMSD between the predicted structures and the input motif. Successful designs were selected using a backbone RMSD cut-off of 1 Å and clustered using single-linkage clustering at a TM-score threshold of 0.6 to identify the number of unique successes. For comparison, we also computed the number of unique successes based on RMSD-CA. We compared these results with results published for Genie 2 and RFdiffusion in ref. 2 using the same evaluation strategy^2^.

### Symmetry editing

To generate symmetric repeat proteins according to a given symmetry group, a representative subunit structure was generated by aligning all the subunits of a repeat protein and averaging their positions. Subunits were aligned using the action of the symmetry group. For cyclic groups, all consecutive subunits were rotated around the symmetry axis onto a single subunit. For a screw (helical) symmetry group, consecutive subunits are first centred along the screw axis and then rotated onto a single subunit around the axis. We can then position the centre of mass of the subunit at a specified radius *R* from the symmetry axis to generate structures with a specified radius. The resulting representative structure is then replicated using the group action. This process is described in detail in Supplementary Algorithm 10 for a group *G* with a single generator *g*.

### Symmetry benchmark

We generated symmetric repeat proteins with subunits of lengths 50 and 100 for cyclic symmetry groups *C*_3_, *C*_4_ and *C*_5_ with variable radii using VP diffusion as well as *C*_3_ to *C*_7_ with radii from 10 Å to 14 Å using VE diffusion. In addition, we generated screw-symmetric designs with 2–3 repeat subunits for various angles and radii. For each design class, we generated 20 symmetrized backbones and designed ten sequences using ProteinMPNN with a temperature of 0.1 (ref. 20). We evaluated designability using ESMFold for all the designs. To verify that the designed screw-symmetric proteins would be predicted to fold with more repeats added, we used AlphaFold 3 (ref. 72) to verify the structure of nine-subunit repeats for a subset of designs.

### Multi-state structure editing

Multi-state outputs were generated by running one independent diffusion process per state and editing the denoised output structures to fix shared substructures across states. To fix a set of residues across states, we aligned the fixed residue positions, optionally averaged them and copied the result back to each state. Repeating this procedure for each denoising step ensures that the fixed residues will have highly similar positions in the final generated structures. Supplementary Algorithm 11 describes the editing process for a two-state design process with a set of fixed residues {*m*}.

### Multi-state design benchmark

We generated designs for the multi-state design problem described in ref. 13 (Supplementary Table III). For each design, three backbones (parent, child 1 and child 2) were generated with secondary structure conditioning according to ref. 13 using the editing strategy described in the ‘Multi-state structure editing’ section. Editing was performed with two different conditions: either the structure of the terminal helices (unconstrained) or the structure of all helices (constrained) shared between parent and children was fixed using structure editing. Here 1,000 designs were generated per condition. We used ProteinMPNN^20^ with a temperature of 0.1 to generate ten sequences for each set of backbones, fixing amino acid identities across the parent and child sequences. To allow a comparison with the results presented in ref. 13, AlphaFold 2 (ref. 23) was used to determine the designability. We used the cut-offs for success (scRMSD < 3 Å and pLDDT > 75) reported in ref. 13.

### Software tools

We used Foldseek v.7.04e0ec8 for structural alignment and clustering^55^. Protein structures were additionally aligned using TM-align v.20220412. For dataset generation, sequences of PDB proteins were clustered using mmseqs2 v.4f046dd1979ec87b440656ff13b12e5c5 25b8374. For structure predictions, novobench used AlphaFold v.2.3.1 and ESMFold v.1.0.3.

### Reporting summary

Further information on research design is available in the Nature Portfolio Reporting Summary linked to this article.

## Extended Data

**Extended Data Fig. 2:**
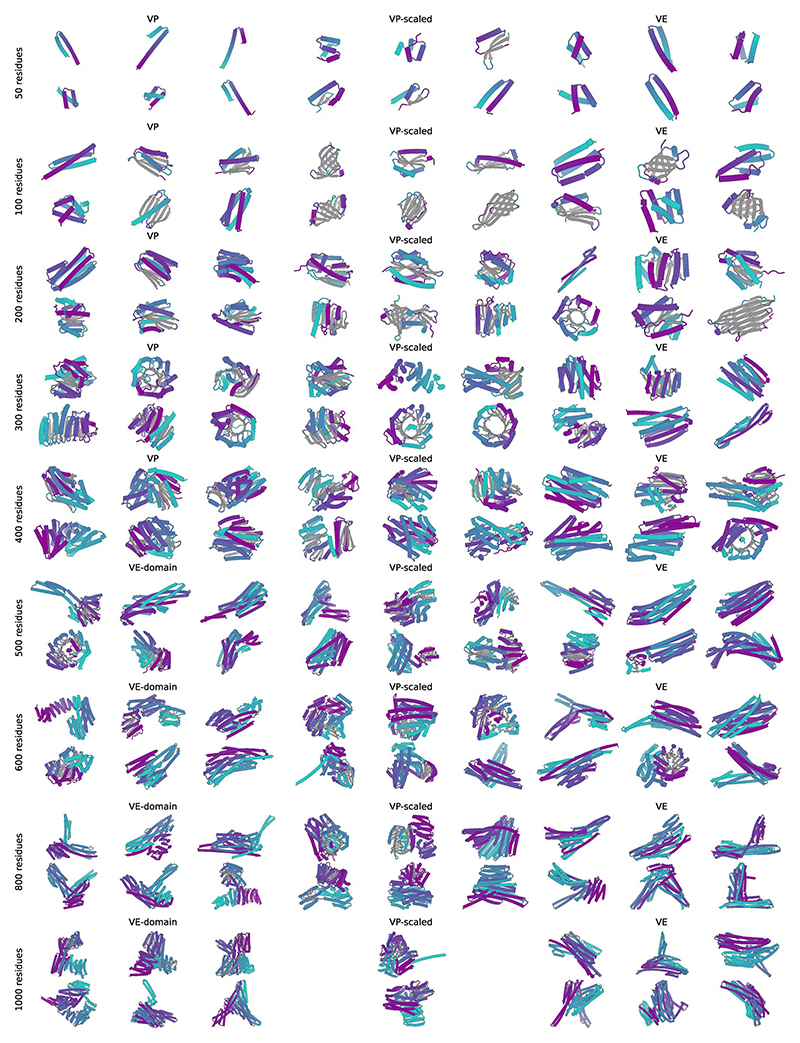
Example unconditional monomer designs. Monomers of length 50, 100, 200, 300, 400, 500, 600, 800 and 1,000 residues, using VP, VP-scaled and VE models with and without domain-like noise. Designs shown are the first up to 6 designs with scRMSD < 2.0 Å and pLDDT > 70 for each length and model type. Structures are coloured by residue index (N-terminus: purple, C-terminus: turquoise) and beta sheets are coloured in grey.

**Extended Data Fig. 3:**
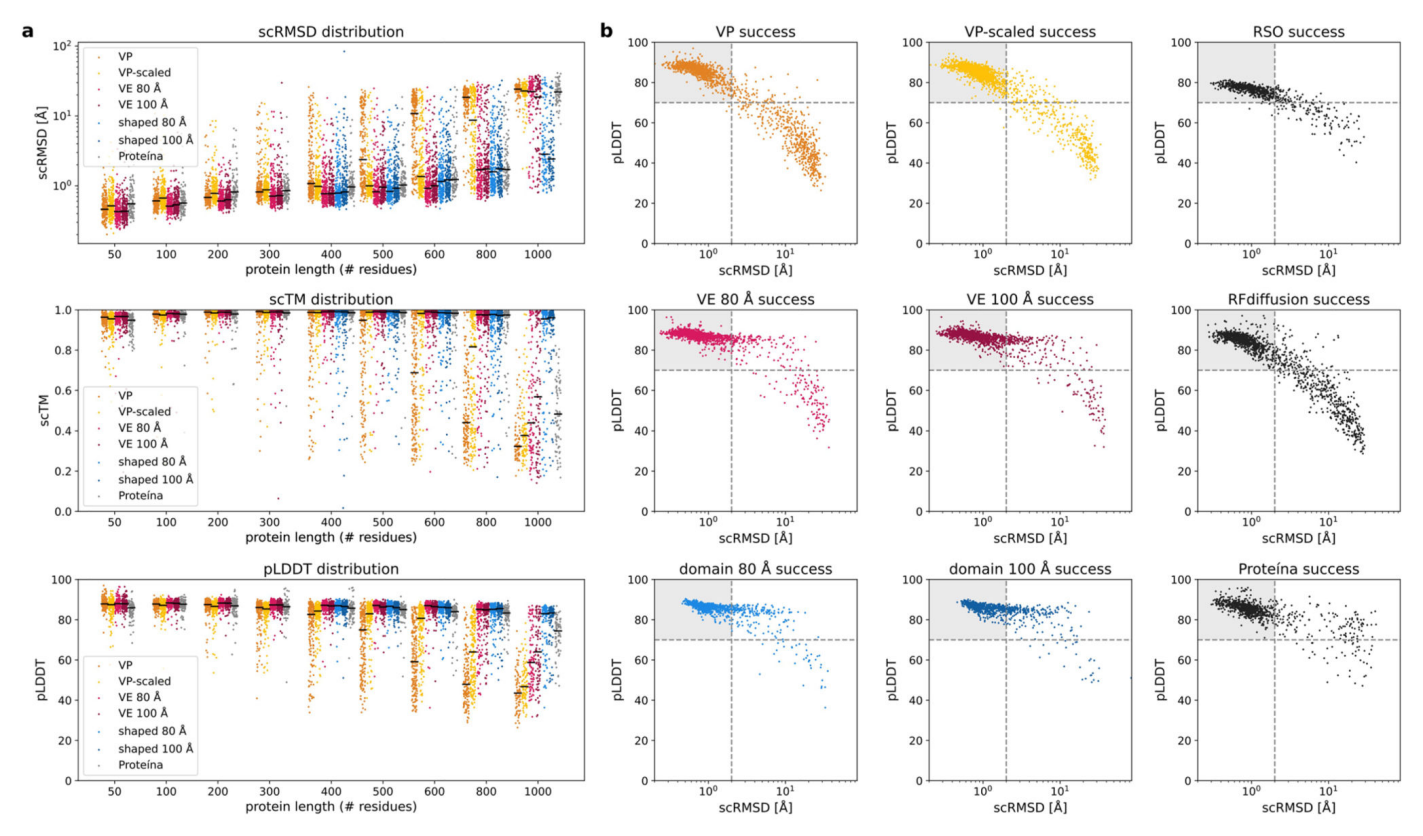
Unconditional monomer designability statistics. (**A**) Distributions of scRMSD, scTM between generated and ESMfold-predicted structures as well as ESMfold pLDDT for each backbone, taking the best value over 8 ProteinMPNN sequences for each backbone. Each point corresponds to a backbone. Points are coloured and grouped by model type and noise schedule. The solid black lines indicate the median value for each model. (**b**) Joint distributions of ESMfold scRMSD and pLDDT over all structures generated with salad models with results for RSO and RFdiffusion shown from comparison (results were obtained from [27]). Scatterplots are coloured as in (**a**). The dashed lines indicate the success cutoff values of scRMSD < 2.0 Å and pLDDT > 70 and the shaded area corresponds to the area of successful designs. Based on n = 200 generated backbones per condition.

**Extended Data Fig. 4:**
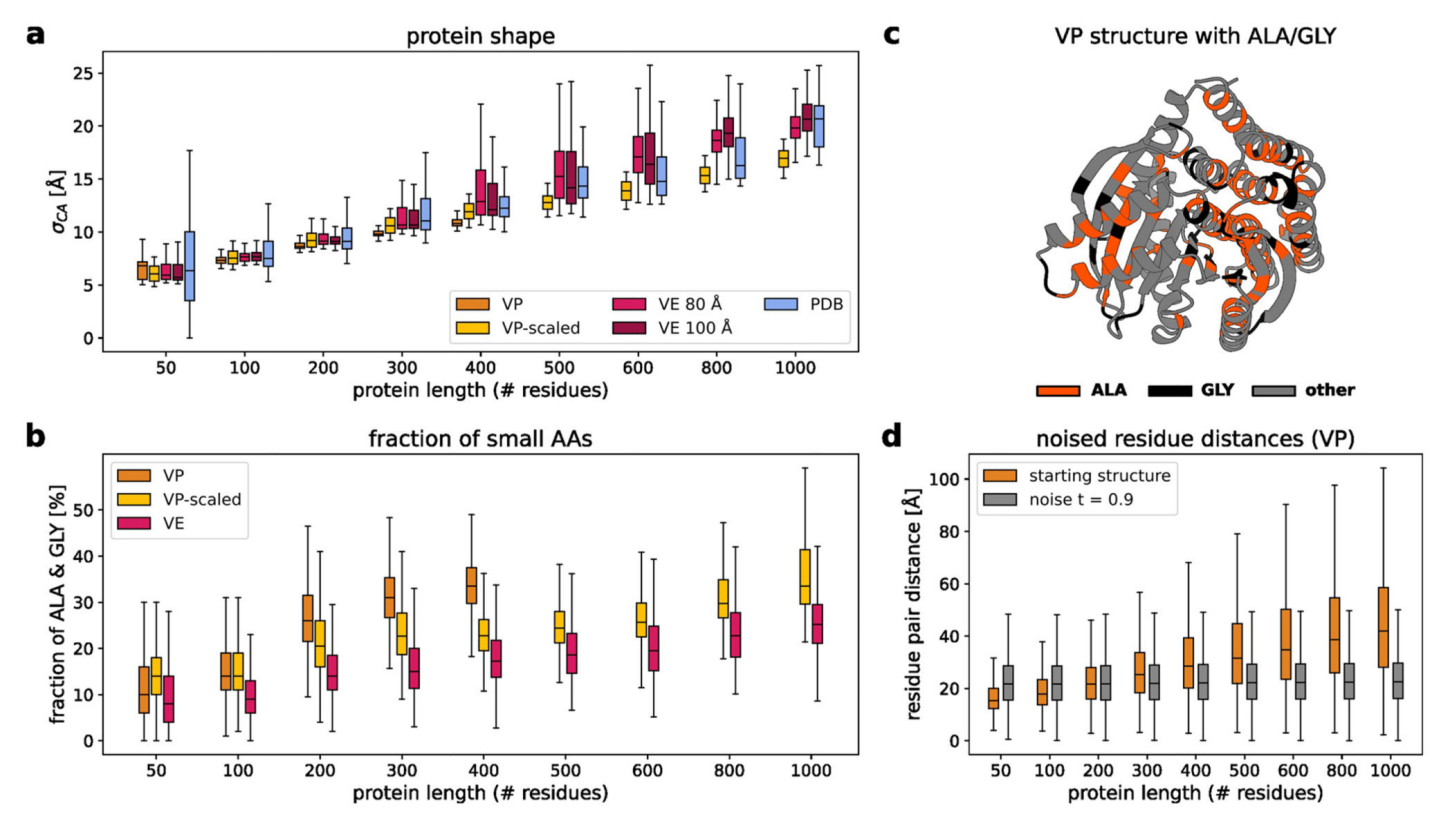
Impacts of VP noise on protein shape and sequence composition. (**a**) Boxplot of CA atom position standard deviations for protein structures in the PDB and backbones generated using different noise schedules. *σ*_CA_ distributions are shown for protein lengths between 50 and 1,000 residues. VP noise consistently produces highly-compact backbones with low *σ*_CA_, while VP-scaled and VE noise result in higher *σ*_CA_ structures more closely matching the *σ*_CA_ of proteins in the PDB. (**b**) Boxplots of the fraction of alanine (ALA) and glycine (GLY) residues in sequence designs for backbones generated using VP, VP-scaled and VE diffusion. A high proportion of ALA and GLY residues in a designed sequence coincides with the presence of tightly-packed secondary structure elements with no space for more bulky amino acids. The fraction of small amino acids increases with protein length for all models but shows a particularly pronounced increase for structures generated using VP noise. In comparison, VP-scaled and VE models show a slower increase and an overall lower fraction of ALA and GLY residues. (**c**) Example structure of a 400 residue backbone generated using VP noise. ALA (GLY) residues are marked in red (black). (**d**) Boxplot of amino acid pair CA distances for proteins of size 50 to 400. Distributions of distances are shown for noise-free structures (orange) and noised structures at diffusion time t = 0.9. At low protein lengths (50 - 200), a VP model needs to decrease CA distances to denoise the structure, whereas at high protein lengths (>= 300 residues) it needs to increase CA distances to arrive at the denoised structure. A VP model mostly trained on smaller proteins will therefore likely develop a bias for lower CA distances resulting in overly compact, undesignable structures. (**a, b, d**) For all box plots, the center line indicates the median, box boundaries the 1st and 3rd quartiles and whiskers 1.5 × the inter-quartile range from the box. Distributions were computed over n = 200 generated backbones for each protein length.

**Extended Data Fig. 5:**
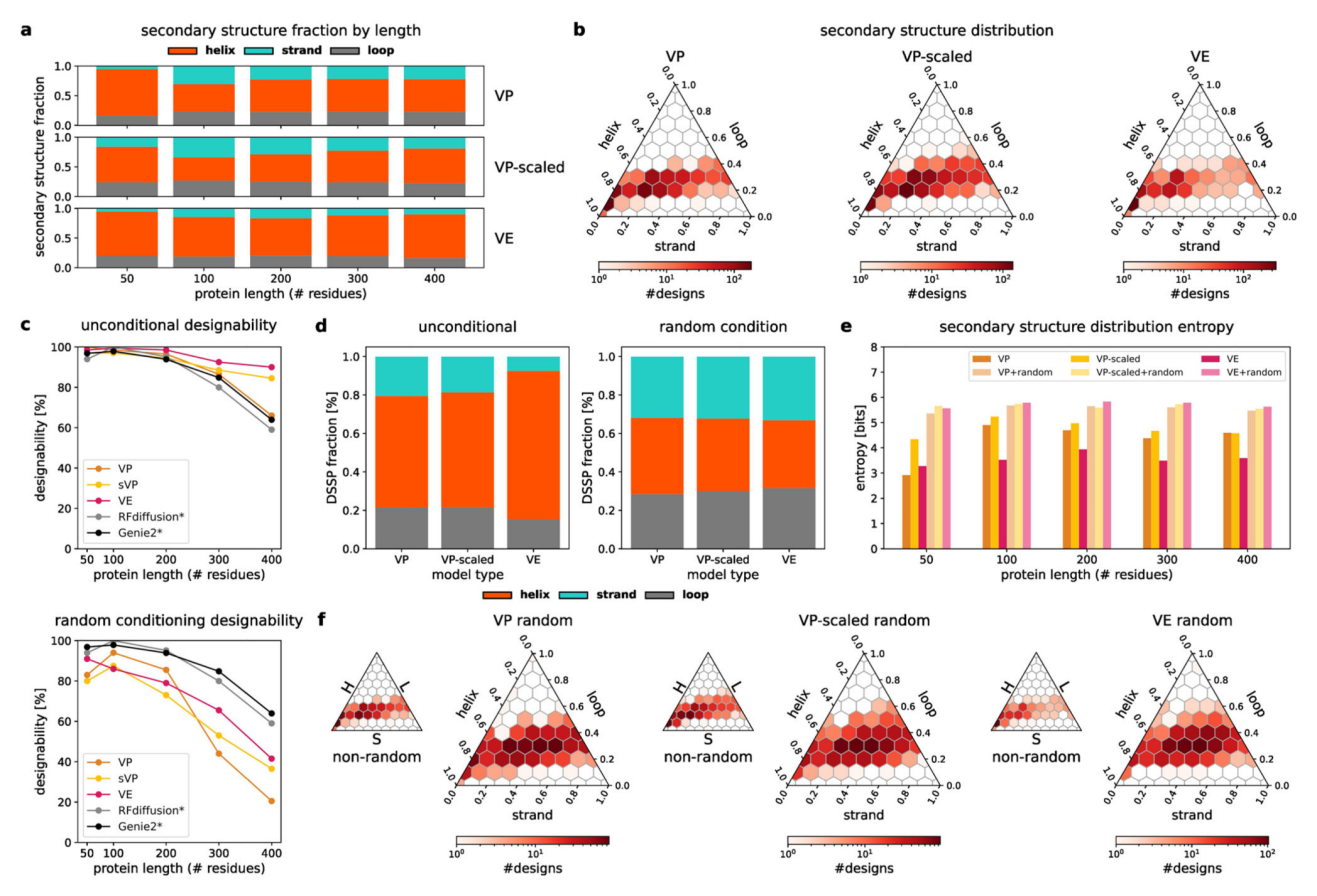
Effects of random secondary structure conditioning on diversity and designability. (**a**) Secondary structure fraction for protein structures between 50 and 400 residues generated using unconditional sampling from salad VP, VP-scaled and VE models. (**b**) Ternary plots of the distribution of secondary structure contents in generated backbones of length 50 to 400 residues using salad VP, VP-scaled and VE. (**c**) Designability of salad VP, VP-scaled (sVP) and VE generated structures for proteins of length 50 to 400 residues compared to results for Genie2 and RFdiffusion. (top) designability for unconditional generation; (bottom) designability for random conditioning for salad models compared to unconditional generation for RFdiffusion / Genie2. (**d**) Overall secondary structure distribution for unconditional (left) and randomly conditioned (right) generations for proteins of length 50 to 400 using salad VP, VP-scaled and VE models. (**e**) Bar graph of binned secondary structure distribution entropy for unconditional and randomly conditioned generations of length 50 to 400. The binned secondary structure distributions were constructed by subdividing the range of helix and strand percentages into 20x20 bins of equal size. (**f**) Ternary plots of the distribution of secondary structure content for all generated structures between 50 and 400 residues using salad VP, VP-scaled and VE models with unconditional sampling (inset) and random conditioning.

**Extended Data Fig. 6:**
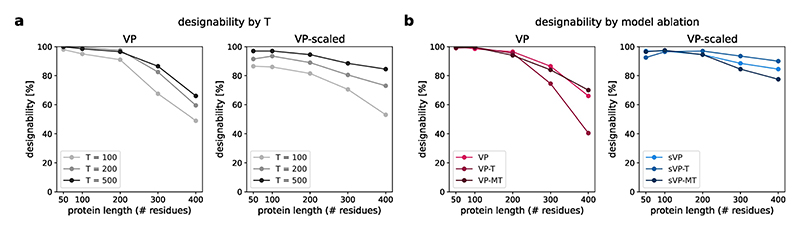
Impact of sampling steps and model architecture on designability. (**a**) ESMfold designability for 8 ProteinMPNN sequences per backbone of length 50 to 400 residues for salad VP and VP-scaled models at 100, 200 or 500 denoising steps. (**b**) ESMfold designability as in (**a**) for salad VP and VP-scaled (sVP) models at 500 denoising steps, as well as variants without diffusion time embedding (-T) and additionally using only minimal distance and orientation pair features (-MT).

**Extended Data Fig. 7:**
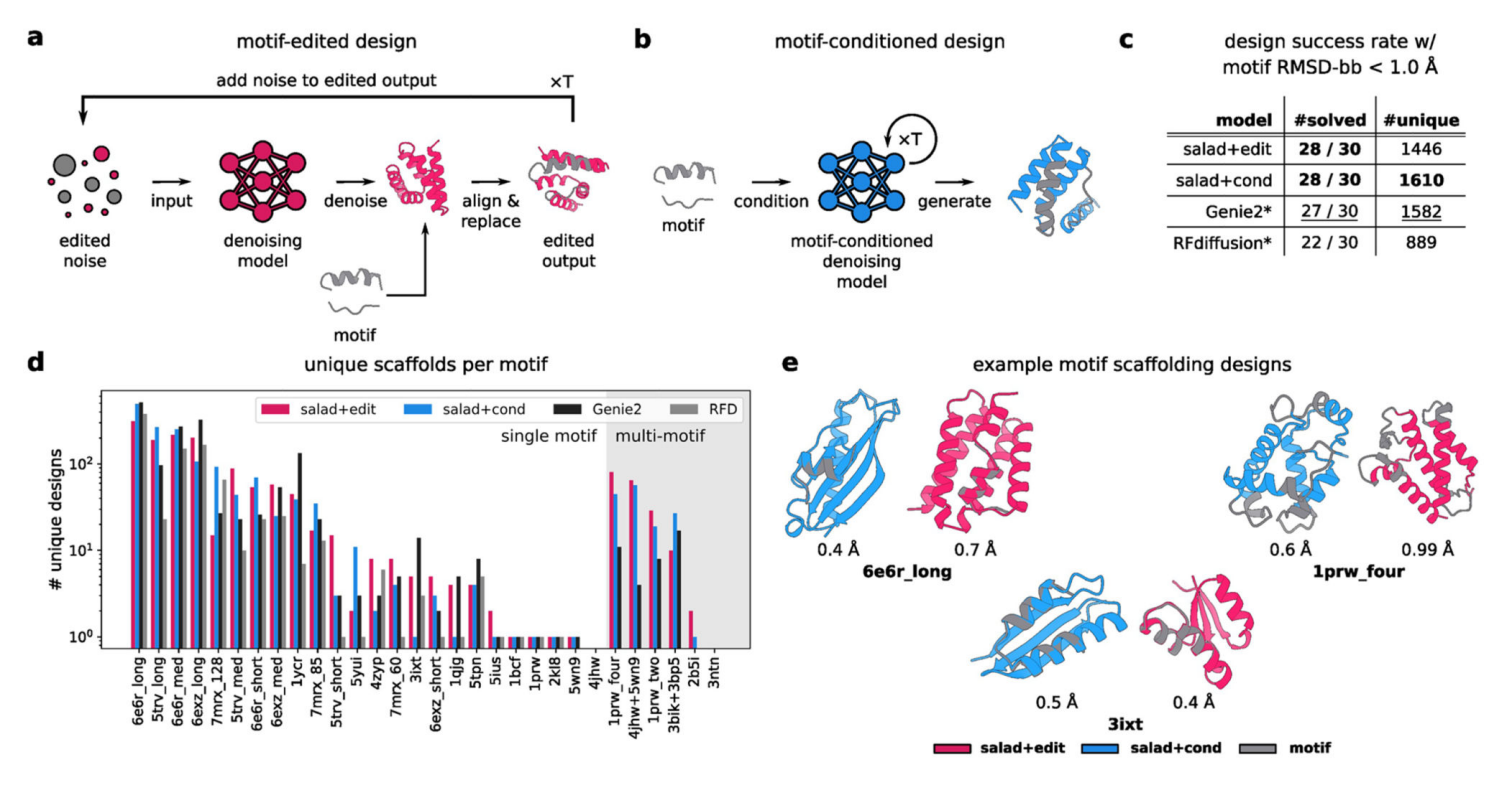
Motif scaffolding. (**a**) Schematic of the output-editing procedure for motif scaffolding. (**b**) Schematic of model conditioning for motif scaffolding. (**c**) Table of unique successful designs on the motif scaffolding benchmark established by Lin et al. 2024^2^ using structure editing and model conditioning. Results for RFdiffusion and Genie2 are shown as reported by Lin et al. 2024^2^. (**d**) Bar plot of the number of unique successful designs (as measured by single-linkage clustering at TM score < 0.6) out of n = 1,000 generated backbones for salad models with editing and conditioning, compared to results reported in Lin et al. 2024. All evaluations were performed with the same settings as Lin et al. 2024^2^. (**e**) Example structures of scaffolded motifs using structure editing and model conditioning. All displayed structures are ESMfold predictions of designed sequences with the motifs marked in grey. Motif RMSD is reported below each structure.

**Extended Data Fig. 8:**
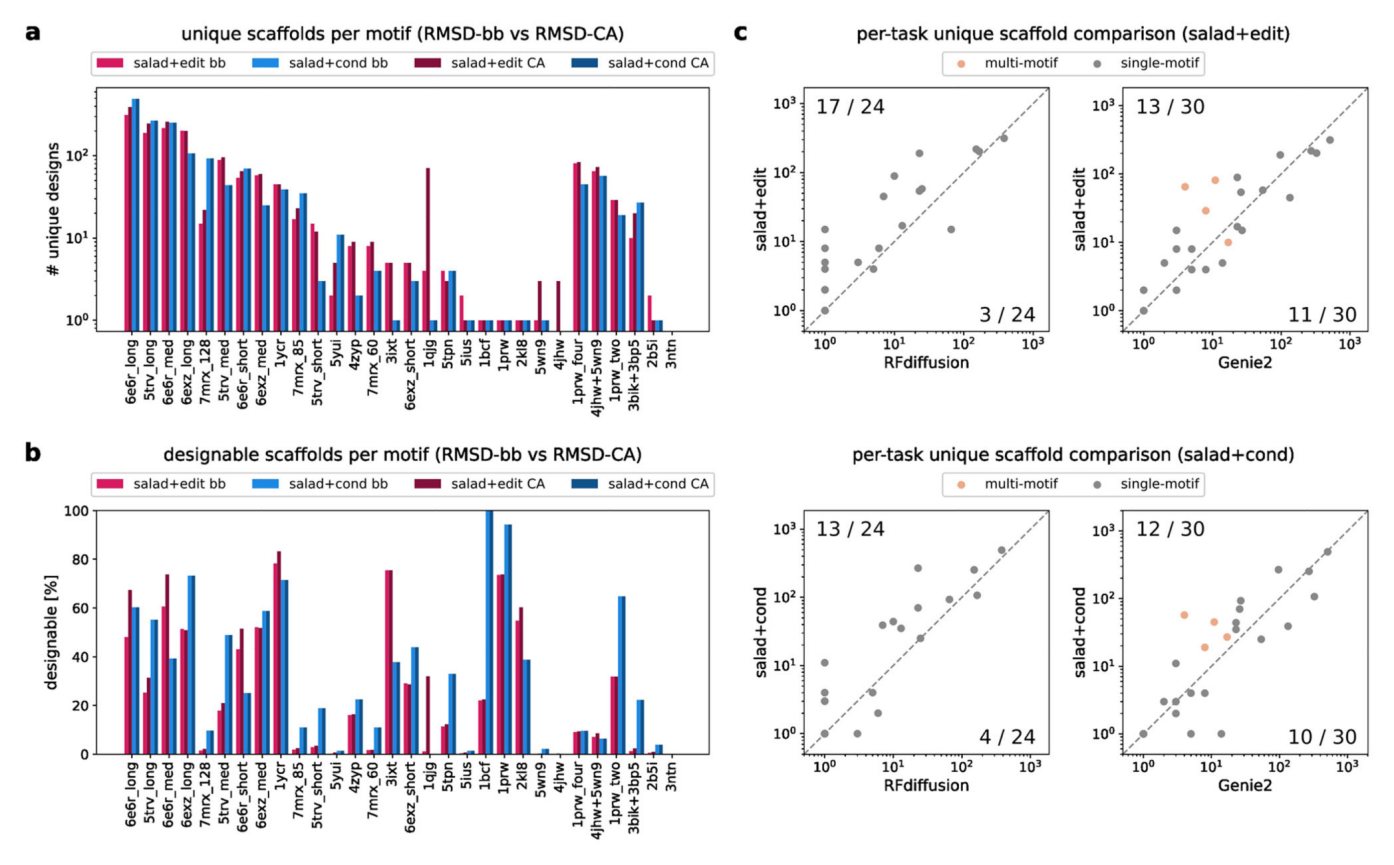
Additional motif scaffolding performance metrics. (**a**) Number of unique scaffolds out of n = 1,000 generated per motif-scaffolding problem using backbone atom (N, CA, C, O) RMSD (bb) and CA RMSD (CA) as a threshold for success. Results are shown for both structure-editing (salad+edit) and motif conditioned models (salad+cond). (**b**) Percentage of successful designs for each motif-scaffolding problem. (**c**) Scatter plots comparing the number of unique successful scaffolds for all single (n = 24) and multi-motif (n = 6) scaffolding tasks between salad models and state-of-the-art diffusion models (Genie2, RFdiffusion). The x and y axes show the number of unique scaffolds for each model. Points on the dashed line correspond to motifs with an equal number of designs for both methods. For points above the line, salad is better; for points below the line RFdiffusion/Genie2 is better. The number of points above and below the line is listed in the upper left and lower right corners.

**Extended Data Fig. 9:**
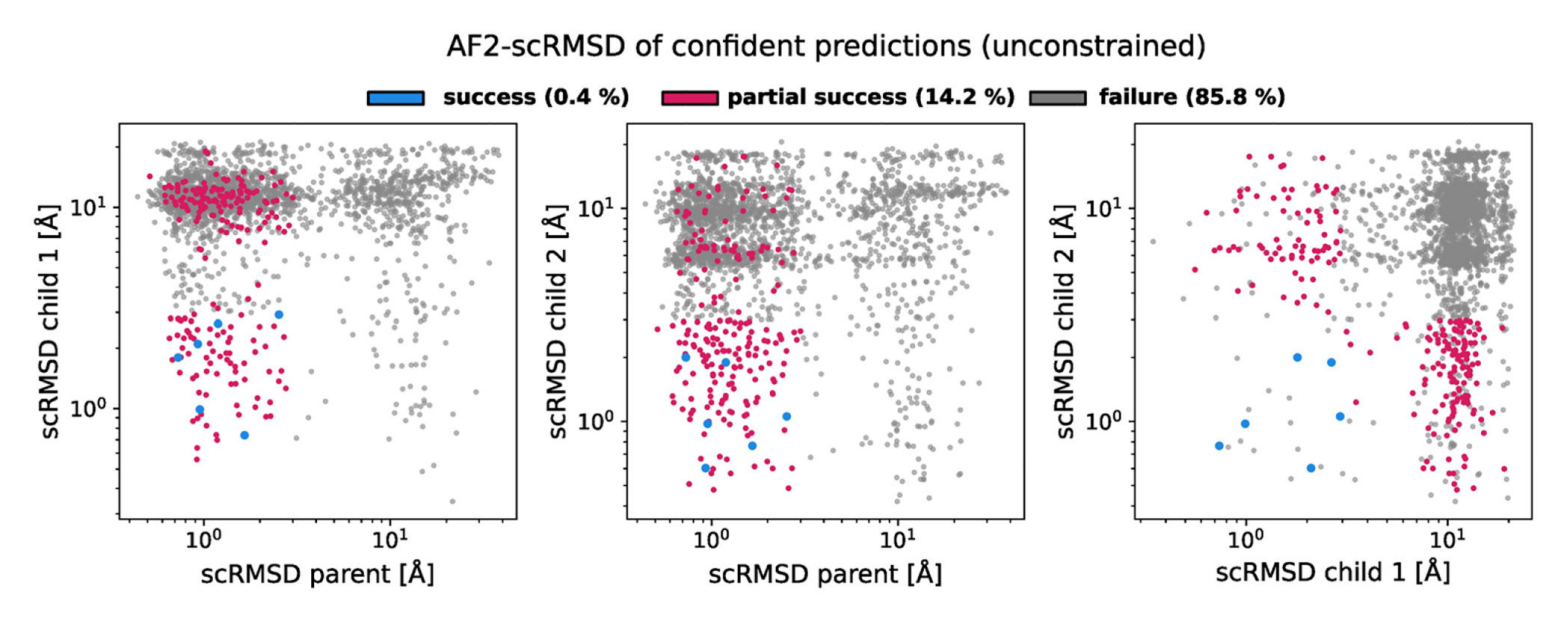
Design success for unconstrained multi-state design. Scatter plots of scRMSD for parent and child designs generated only with secondary structure conditioning, without fixing parts of the structure across denoising processes. Only designed sequences with AF2-pLDDT > 75 are shown. Successful designs (0.4 % of backbones) are shown in blue, partially successful designs (14.2 %) in red and failed designs (85.8 %) in grey. n = 1,000 structures generated and evaluated per condition.

**Extended Data Table 1:** Diffusion model hyperparameters and parameter counts

model	sparse	# layers	# AA features	# pair features	# parameters
salad (Encoder + Diffusion + AADecoder)	yes	2 + 6 + 3	128	64	**11.9M**
salad (Diffusion)	yes	6	128	64	**8.4M**
Chroma	yes	12	512	256	18.5M
Genie 2	no	8	384	128	15.7M
RFdiffusion	no	36	256	128	59.8M
Proteína	no	15	768	512	200M

## Supplementary Material

**Supplementary information** The online version contains supplementary material available at https://doi.org/10.1038/s42256-025-01100-z.

Supplementary Information (Supplementary Algorithms 1–11 and Tables I–III.)

Nature Portfolio Reporting Summary

## Data Availability

The generated protein structures and ESMFold-based scores and structure predictions from this work have been deposited in Zenodo (https://zenodo.org/records/14711580)^73^, which also contains the parameters of models used in this study and a snapshot of the source code that was used to generate them. The source code on Zenodo (https://zenodo.org/records/14711580)^73^ and GitHub (https://github.com/mjendrusch/salad) contains instructions and scripts to reconstruct the datasets used for training in this study, as well as the training scripts used to produce the model parameters.

## Code availability

The code for all models described in this work is available under an Apache 2.0 license via GitHub at github.com/mjendrusch/salad. Parameters for those models are available under a CC BY 4.0 license via GitHub at github.com/mjendrusch/salad and via Zenodo (https://zenodo.org/records/14711580)^73^. The code for the AlphaFold 2- and ESMFold-based benchmarking is available under an Apache 2.0 license via GitHub at github.com/mjendrusch/novobench.

## References

[1] Watson JL (2023). De novo design of protein structure and function with RFdiffusion. Nature.

[2] Lin Y, Lee M, Zhang Z, AlQuraishi M (2024). Out of many, one: designing and scaffolding proteins at the scale of the structural universe with Genie 2.

[3] Zorine D, Baker D (2024). De novo design of alpha-beta repeat proteins. bioRxiv.

[4] Brunette TJ (2015). Exploring the repeat protein universe through computational protein design. Nature.

[5] Wicky BIM (2022). Hallucinating symmetric protein assemblies. Science.

[6] Wang J (2022). Scaffolding protein functional sites using deep learning. Science.

[7] Castro KM (2024). Accurate single domain scaffolding of three non-overlapping protein epitopes using deep learning. bioRxiv.

[8] Bennett NR (2024). Atomically accurate de novo design of single-domain antibodies. bioRxiv.

[9] Glögl M (2024). Target-conditioned diffusion generates potent TNFR superfamily antagonists and agonists. Science.

[10] Pacesa M (2024). BindCraft: one-shot design of functional protein binders. bioRxiv.

[11] Praetorius F (2023). Design of stimulus-responsive two-state hinge proteins. Science.

[12] Pillai A (2024). De novo design of allosterically switchable protein assemblies. Nature.

[13] Lisanza SL (2025). Multistate and functional protein design using RoseTTAFold sequence space diffusion. Nat Biotechnol.

[14] Richter F, Leaver-Fay A, Khare SD, Bjelic S, Baker D (2011). De novo enzyme design using Rosetta3. PLoS ONE.

[15] Hsien-Wei Yeh A (2023). De novo design of luciferases using deep learning. Nature.

[16] Lauko A (2025). Computational design of serine hydrolases. Science.

[17] Sesterhenn F (2020). De novo protein design enables the precise induction of RSV-neutralizing antibodies. Science.

[18] Correia BE (2014). Proof of principle for epitope-focused vaccine design. Nature.

[19] Krishna R (2024). Generalized biomolecular modeling and design with RoseTTAFold All-Atom. Science.

[20] Dauparas J (2022). Robust deep learning based protein sequence design using ProteinMPNN. Science.

[21] Ingraham J (2022). Illuminating protein space with a programmable generative model. Nature.

[22] Akpinaroglu D (2023). Structure-conditioned masked language models for protein sequence design generalize beyond the native sequence space. bioRxiv.

[23] Jumper JM (2021). Highly accurate protein structure prediction with AlphaFold. Nature.

[24] Wu R Min (2022). High-resolution de novo structure prediction from primary sequence. bioRxiv.

[25] Mariani V, Biasini M, Barbato A, Schwede T (2013). lDDT: a local superposition-free score for comparing protein structures and models using distance difference tests. Bioinformatics.

[26] Lin Y, AlQuraishi M, Krause A (2023). Generating novel, designable, and diverse protein structures by equivariantly diffusing oriented residue clouds.

[27] Frank C (2024). Scalable protein design using optimization in a relaxed sequence space. Science.

[28] Bennett NR (2022). Improving de novo protein binder design with deep learning. Nat Commun.

[29] Zhang Y, Skolnick J (2005). TM-align: a protein structure alignment algorithm based on the TM-score. Nucleic Acids Res.

[30] Leaver-Fay A (2011). Rosetta3: an object-oriented software suite for the simulation and design of macromolecules. Methods Enzymol.

[31] Anishchenko I (2021). De novo protein design by deep network hallucination. Nature.

[32] Jendrusch MA (2025). AlphaDesign: a de novo protein design framework based on AlphaFold. Mol Syst Biol.

[33] Goudy OJ, Nallathambi A, Kinjo T, Randolph NZ, Kuhlman B (2023). In silico evolution of autoinhibitory domains for a PD-L1 antagonist using deep learning models. Proc Natl Acad Sci USA.

[34] Bryant P, Elofsson A (2022). EvoBind: in silico directed evolution of peptide binders with AlphaFold. bioRxiv.

[35] Ho J, Jain A, Abbeel P (2020). Denoising diffusion probabilistic models. Adv Neural Inf Process Syst.

[36] Alamdari S (2023). Protein generation with evolutionary diffusion: sequence is all you need. bioRxiv.

[37] Wang C, Salakhutdinov R (2024). Proteus: exploring protein structure generation for enhanced designability and efficiency.

[38] Yim J, Krause A (2023). SE(3) diffusion model with application to protein backbone generation.

[39] Baek M (2021). Accurate prediction of protein structures and interactions using a three-track neural network. Science.

[40] Chu AE (2024). An all-atom protein generative model. Proc Natl Acad Sci USA.

[41] Lee JS, Kim J, Kim PM (2023). Score-based generative modeling for de novo protein design. Nat Comput Sci.

[42] Geffner T, Yue Y (2025). Proteina: scaling flow-based protein structure generative models.

[43] Vaswani A (2017). Attention is all you need. Adv Neural Inf Process Syst.

[44] Ingraham J, Garg V, Barzilay R, Jaakkola T (2019). Generative models for graph-based protein design. Adv Neural Inf Process Syst.

[45] Nguyen TQ, Salazar J, Niehues J (2019). Transformers without tears: improving the normalization of self-attention.

[46] Shazeer N (2020). GLU variants improve transformer.

[47] Berman HM (2002). The protein data bank. Acta Cryst.

[48] Van Den Oord A, Vinyals O, Kavukcuoglu K (2017). Neural discrete representation learning. Adv Neural Inf Process Syst.

[49] Hayes T (2025). Simulating 500 million years of evolution with a language model. Science.

[50] Kryshtafovych A, Schwede T, Topf M, Fidelis K, Moult J (2021). Critical assessment of methods of protein structure prediction (CASP)—round XIV. Proteins.

[51] Kryshtafovych A, Schwede T, Topf M, Fidelis K, Moult J (2023). Critical assessment of methods of protein structure prediction (CASP)—round XV. Proteins.

[52] Frank C, Schiwietz D, Fuß L, Ovchinnikov S, Dietz H (2024). AlphaFold2 refinement improves designability of large de novo proteins. bioRxiv.

[53] Apic G, Gough J, Teichmann SA (2001). Domain combinations in archaeal, eubacterial and eukaryotic proteomes. J Mol Biol.

[54] Varadi M (2024). AlphaFold protein structure database in 2024: providing structure coverage for over 214 million protein sequences. Nucleic Acids Res.

[55] Van Kempen M (2024). Fast and accurate protein structure search with Foldseek. Nat Biotechnol.

[56] Campbell A, Yim J, Barzilay R, Rainforth T, Jaakkola T, Salakhutdinov R (2024). Generative flows on discrete state-spaces: enabling multimodal flows with applications to protein co-design.

[57] Liu Y, Wang S, Liu H (2024). De novo protein design with a denoising diffusion network independent of pretrained structure prediction models. Nat Methods.

[58] Madhurima K, Nandi B, Sekhar A (2021). Metamorphic proteins: the Janus proteins of structural biology. Open Biol.

[59] Martin A, Berner CA, Ovchinnikov S, Vorobieva AA (2024). Validation of de novo designed water-soluble and transmembrane proteins by in silico folding and melting. Protein Sci.

[60] Barrio-Hernandez I (2023). Clustering predicted structures at the scale of the known protein universe. Nature.

[61] Chu AE (2024). An all-atom protein generative model. Proc Natl Acad Sci USA.

[62] Anand N, Achim T (2022). Protein structure and sequence generation with equivariant denoising diffusion probabilistic models.

[63] Qu W (2024). P (all-atom) is unlocking new path for protein design. bioRxiv.

[64] Nichol AQ, Dhariwal P, Meila M, Zhang T (2021). Improved denoising diffusion probabilistic models.

[65] Karras T, Aittala M, Aila T, Laine S (2022). Elucidating the design space of diffusion-based generative models. Adv Neural Inf Process Syst.

[66] Li X, Thickstun J, Gulrajani I, Liang PS, Hashimoto TB (2022). Diffusion-LM improves controllable text generation. Adv Neural Inf Process Syst.

[67] Hoogeboom E, Hofmann K (2022). Autoregressive diffusion models.

[68] Xiong R, Chauduri K (2020). On layer normalization in the transformer architecture.

[69] Steinegger M, Söding J (2017). MMseqs2 enables sensitive protein sequence searching for the analysis of massive data sets. Nat Biotechnol.

[70] Kingma DP, Ba J, Larochellem H (2015). Adam: a method for stochastic optimization.

[71] Loshchilov I, Hutter F, Bengio Y (2017). SGDR: stochastic gradient descent with warm restarts.

[72] Abramson J (2024). Accurate structure prediction of biomolecular interactions with AlphaFold 3. Nature.

[73] Jendrusch M, Korbel J (2025). Raw data for ‘efficient protein structure generation with sparse denoising models’.

[74] European Molecular Biology Laboratory (2024). Embl heidelberg hpc cluster.

