# Supplementary material for: Efficient protein structure generation with sparse denoising models
Source: Nat Mach Intell. Author manuscript; Available in PMC 2025 Nov 27. (PMC7618410; doi:10.1038/s42256-025-01100-z)
Supplement: Supplementary Information (Supplementary Algorithms 1–11 and Tables I–III.) [file EMS210903-supplement-Supplementary_Information__Supplementary_Algorithms_1_11_and_Tables_I_III__.pdf]

nature machine intelligence

Supplementary information https://doi.org/10.1038/s42256-025-01100-z

Efficient protein structure generation with sparse denoising models

In the format provided by the authors and unedited

Supplementary Information for "Efficient protein structure generation with sparse denoising models"

CONTENTS

Algorithms

1 Denoising model inference 2
2 Denoising module 3
3 neighbour selection 3
4 Residue pair features 4
5 Lightweight global update 4
6 Position update 4
7 Per-block distogram prediction. 5
8 Distogram attention. 5
9 Motif editing denoising process 5
10 Symmetry-group editing 6
11 Multi-state editing 6

Supplementary Tables

I Default model settings for runtime benchmark 7
II Secondary structure element parameters. 7
III Multi-state secondary structure conditioning 7

ALGORITHMS

Algorithm 1: Denoising model inference

```
// sample a protein structure x with condition c using a denoising model
// applying self-conditioning for t < t_prev and returning a structure at t_exit
// using structure-editing with edit_noise and edit_out
def denoising_model_inference(c, t_prev, t_exit; edit_noise, edit_out):
    // initialize positions
    x = 0;
    x_prev = x;
    // run denoising process
    for t in (t_N, ..., t_0) do
        // apply noise
        x_t = apply_noise_t(x);
        // optionally edit noise
        x_t = edit_noise(x_t);
        // apply the model to produce a denoised structure
        x = salad(x_t; σ_t, x_prev, c);
        x_prev = x;
        // disable self-conditioning for later time-steps
        if t < t_prev then
            x_prev = 0;
        // optionally edit the denoised structure
        x = edit_out(x);
        // return structure at non-zero noise level
        if t ≤ t_exit then
            return x
    return x
```

Algorithm 2: Denoising module

```
// denoises a set of residue positions x and updates residue representation local_i
def DenoisingModule(local_i, x, x_prev, r, d_cond, m_cond, b_ij):
    trajectory = ();
    repeat
        local_i, x = DenoisingBlock(local_i, x, x_prev, r, d_cond, m_cond, b_ij);
        trajectory = concatenate(trajectory, x);
    until for 6 blocks;
    return local_i, trajectory

// single denoising block which equivariantly updates x
def DenoisingBlock(local_i, x, x_prev, r, d_cond, m_cond, b_ij):
    // select amino acid neighbours for current positions
    neighbors_ij = get_neighbors(x, r, d_cond, m_cond, b_ij);        int N × K; K = 48
    x_j, r_j = x[neighbors_ij], r[neighbors_ij];
    // compute pair features using current positions
    pair_ij = pair_features(x_i, x_j, r_i, r_j, d_cond, m_cond, b_ij);        float N × K × pair_size
    // compute sparse attention with current positions
    local_i += SparseIPA(LayerNorm(local_i), pair_ij);        float N × local_size
    // select amino acid neighbours for previous positions
    neighbors_ij = get_neighbors(x_prev, r, d_cond, m_cond, b_ij);
    x_j, r_j = x[neighbors_ij], r[neighbors_ij];
    // compute pair features using previous positions
    pair_ij = pair_features(x_i, x_j, r_i, r_j, d_cond, m_cond, b_ij);
    // compute sparse attention with current positions
    local_i += SparseIPA(LayerNorm(local_i), pair_ij);
    // GeLU gated MLP update
    local_i += Update(LayerNorm(local_i));
    // update positions using local features
    x_i = position_update(x_i, local_i);
    return local_i, x_i
```

Algorithm 3: neighbour selection

```
// select neighbours of a residue
def get_neighbors(x, r, d_cond, m_cond, b_ij):
    // select #residue_index nearest neighbours based on residue index distance
    neighbors_ij = KNN_#residue_index(|r_i − r_j|);        int, N × #residue_index
    // select #distance nearest neighbours based on CA distance
    neighbors_ij = KNN_#distance(||x_i − x_j||_2, neighbors_ij);        int, N × (#residue_index + #distance)
    // select #distance nearest neighbours at random with p ∝ 1/d^3
    log p_ij = −3 · log(||x_i − x_j||_2);        float, N × N
    w_ij = log p_ij − g_ij; g_ij ~ Gumbel(0, 1);
    neighbors_ij = KNN_#random(−w_ij, neighbors_ij);        int, N × (... + #random)
    // select additional neighbours with nonzero block condition b_ij
    neighbors_ij = KNN_#cond(−b_ij, neighbors_ij);        int, N × (... + #cond)
    return neighbors_ij

// K-nearest neighbour computation excluding existing neighbours
def KNN_K(d, neighbors_ij):
    // set distances of existing neighbours to infinity to exclude them
    if neighbors_ij exist then
        d[neighbors_ij] = ∞
    // return indices of the K nearest neighbours
    return concatenate_j(neighbors_ij, argsort_j(d_ij)[:, : K])
```

Algorithm 4: Residue pair features

// compute pair features $\texttt{pair}_{ij}$ for a pair of amino acid residues i, j

def $\texttt{pair\_features}$*(x_i, x_j, d_{cond}, m_{cond}, b_{ij}, r_i, r_j, mode (full, minimal))*:

  // residue index distance features

  $\texttt{pair}_{ij} = \texttt{LinearNoBias}(\texttt{one\_hot}(\texttt{clip}(r_i - r_j, -32, 32)))$; $N \times K \times \texttt{pair\_size}$; $\texttt{pair\_size} = 64$

  // distance rbf features for backbone atoms

  $\texttt{pair}_{ij} \mathrel{+}= \texttt{LinearNoBias}(\texttt{distance\_rbf}(||x_{ia} - x_{jb}||_2))$; $N \times K \times \texttt{pair\_size}$

  // relative rotation features

  $\texttt{pair}_{ij} \mathrel{+}= \texttt{LinearNoBias}(\texttt{flatten}(\mathbf{R}_j \cdot \mathbf{R}_i^{-1}))$; $N \times K \times \texttt{pair\_size}$

  // skip direction and pair-vector features in minimal models

  if *mode* **is** *full* **then**

    // directions from CA of residue i to all neighbour atoms

    $\texttt{pair}_{ij} \mathrel{+}= \texttt{LinearNoBias}(\texttt{flatten}(\frac{\mathbf{T}_i^{-1} \cdot x_j}{||\mathbf{T}_i^{-1} \cdot x_j||_2}))$; $N \times K \times \texttt{pair\_size}$

    // atom positions of residue pair in the frame of residue i

    $\texttt{pair}_{ij} \mathrel{+}= \texttt{LinearNoBias}(\texttt{flatten}_{ab}(\mathbf{T}_i^{-1} \cdot \texttt{concatenate}(x_{ia}, x_{jb})))$; $N \times K \times \texttt{pair\_size}$

  // CA distance map conditioning features

  $\texttt{pair}_{ij} \mathrel{+}= m_{cond} \cdot \texttt{LinearNoBias}(\texttt{distance\_rbf}(d_{cond}))$; $N \times K \times \texttt{pair\_size}$

  // chain contact and block contact features

  $\texttt{pair}_{ij} \mathrel{+}= \texttt{LinearNoBias}((b_{ij})$; $N \times K \times \texttt{pair\_size}$

  return $\texttt{LayerNorm}(\texttt{pair}_{ij})$

// compute Gaussian RBF of distances d_{ij}

def $\texttt{distance\_rbf}(d_{ij})$:

  return $\texttt{flatten}_{abk}(\exp(-(d_{iajb} - c_k)^2/\sigma^2))$

Algorithm 5: Lightweight global update

// update $\texttt{local}_i$ for an amino acid

def $\texttt{Update}$*($\texttt{local}_i, x_i$)*:

  // transform x_i to the local frame and project

  $y_i = x_i$ transformed to the local frame; $N \times 20 \times 3$

  // update residue scalar features with residue vector features

  $\texttt{local}_i \mathrel{+}= \texttt{LinearNoBias}(\texttt{local\_size})(\texttt{gelu}(\texttt{LinearNoBias}(2 \cdot \texttt{local\_size})(\texttt{flatten}(y_i))))$;

  // compute gates for local, chain and complex features

  $g_{local,i}, g_{chain,i}, g_{cpx,i} = \texttt{gelu}(\texttt{LinearNoBias}(\texttt{local}_i))$; $N \times 4 \cdot \texttt{local\_size}$

  // hidden state with factor 4

  $h_i = \texttt{LinearNoBias}(\texttt{local}_i)$; $N \times 4 \cdot \texttt{local\_size}$

  // gate hidden states for local, chain-pooled and complex-pooled features

  $h_{local,i} = g_{local} \cdot h$;

  $h_{chain,i} = \texttt{mean}_{j \in \texttt{chain}(i)}(g_{chain,j} \cdot h_j)$;

  $h_{cpx,i} = \texttt{mean}_j(g_{cpx,j} \cdot h_j)$;

  // return zero-initialized projection of the sum of all pooled features

  return $\texttt{LinearNoBias}(h_{local,i} + h_{chain,i} + h_{cpx,i})$; $N \times \texttt{local\_size}$

Algorithm 6: Position update

// update x_i for each residue

def $\texttt{position\_update}$*($\texttt{local}_i, x_i$)*:

  // transform x_i to the local frame

  $y_i = x_i$ transformed to the local frame;

  // project local features to positions

  $u_i = \texttt{LinearNoBias}(\texttt{local}_i)$; $N \times N_{atoms} \times 3$

  // update positions in the local frame

  $y_i = y_i + u_i$;

  return y_i *transformed to global coordinates*

Algorithm 7: Per-block distogram prediction.

// light-weight distogram prediction to compute additional neighbours

def $\texttt{BlockDistogram}(\texttt{local}_i)$**:**

  // compute query and key features

  $q_{iad} = \texttt{gelu}(\texttt{LinearNoBias}(\texttt{local}_i));$ $N \times 8 \times 16$

  $k_{jbd} = \texttt{LinearNoBias}(\texttt{local}_j);$ $N \times 8 \times 16$

  // project outer product with weights $\mathbf{W}$ to compute distogram logits

  $\texttt{logits}_{ijd} = \sum_{ab} \mathbf{W}_{abd} q_{iad} k_{jbd};$ $N \times N \times 16$

  // return normalized distogram logits

  return $\texttt{log\_softmax}(\texttt{logits}_{ijd})$

Algorithm 8: Distogram attention.

// sparse attention using distogram neighbours

def $\texttt{DistogramIPA}(\texttt{local}_i)$**:**

  // compute distogram logits, probabilities and mean

  $\texttt{logits}_{ijd} = \texttt{BlockDistogram}(\texttt{local}_i);$

  $p_{ijd} = \texttt{softmax}_d(\texttt{logits}_{ijd});$

  $d^*_{ij} = \sum_d \texttt{bin\_center}_d p_{ijd};$

  // get 32 nearest neighbours using the predicted distances d^*

  $\texttt{neighbours}_{ij} = \texttt{KNN}_{32}(d^*_{ij});$

  $x_j, r_j = x[\texttt{neighbors}_{ij}], r[\texttt{neighbors}_{ij}];$

  // compute pair features using positions and predicted distances d^*

  $\texttt{pair}_{ij} = \texttt{pair\_features}(x_i, x_j, r_i, r_j, d^*_{ij});$

  // compute sparse attention

  $\texttt{local\_update}_i = \texttt{SparseIPA}(\texttt{LayerNorm}(\texttt{local}_i), \texttt{pair}_{ij});$

  return $\texttt{local\_update}_i$

Algorithm 9: Motif editing denoising process

// scaffolding a motif m by editing the model output

def $\texttt{edit\_motif}(m)$**:**

  // initialize positions

  $x = 0;$

  // run denoising process

  for step *in 0 .. 400* **do**

    // compute cosine-schedule standard deviation at the current step

    $t = \texttt{step}/500;$

    $\sigma_t = \texttt{cosine\_schedule}(t);$

    // apply VP noise

    $x_t \sim \mathcal{N}(\sqrt{1 - \sigma_t^2} \cdot x, (\sigma_t \cdot 10\text{Å})^2);$

    // denoise structure

    $x = \texttt{salad}(x_t, \sigma_t);$

    // align the motif onto the denoised structure

    $m =$ motif m aligned to x;

    // replace the motif in the denoised structure

    $x_i = m_i$ if i belongs to the motif else x_i;

  return x

Algorithm 10: Symmetry-group editing

```
// generating a repeat protein with N subunits with symmetry defined by the generator g ∈ G
def edit_symmetry(g, N):
    // initialize positions
    x = 0;
    // run denoising process
    for t in time steps do
        // apply noise
        x_t = apply_noise_t(x);
        // symmetrize noise
        x_t = replicate(x_t);
        // denoise
        x = salad(x_t, σ_t);
        // construct average representative structure
        for i in N do
            μ += g^{-i} · get_subunit(x, i);
        μ /= N;
        // optionally move μ to a radius R from the origin
        μ = translate_R(μ);
        // replicate representative structure
        x = replicate(μ, g, N)
    return x
def replicate(μ, g, N):
    x = ();
    for i in N do
        x = concatenate(x, g^i · μ);
    return x
```

Algorithm 11: Multi-state editing

```
// fixing a set of residues {m} across multiple denoising processes
def edit_multi_state(m):
    // initialize positions for each state
    x_0, x_1 = 0, 0;
    // run denoising process
    for t in time steps do
        // apply noise
        x_{0,t} = apply_noise_t(x_0);
        x_{1,t} = apply_noise_t(x_1);
        // denoise all states
        x_0 = salad(x_{0,t}, σ_t);
        x_1 = salad(x_{1,t}, σ_t);
        // extract the fixed substructure
        y_0 = x_0 for residues in {m};
        y_1 = x_1 for residues in {m};
        // align residues
        y'_0 = align y_0 to y_1;
        y'_1 = align y_1 to y_0;
        // option 1: average residue positions and replace
        x_0 = (y_0 + y'_1) / 2 for residues in {m};
        // option 2: directly replace structure of one state with another
        x_1 = y'_0 for residues in {m};
    return x_0, x_1
```

SUPPLEMENTARY TABLES

TABLE I. Default model settings for runtime benchmark

model	# steps
salad	400
Genie2	1,000
RFdiffusion	50
Chroma	500
Proteína	400

TABLE II. Secondary structure element parameters.

structure	min count	max count	min length
helix	$\min(1, \#\texttt{helix}/6)$	$\#\texttt{helix}/6$	6
strand	$\min(2, \#\texttt{strand}/8)$	$\#\texttt{strand}/4$	4

TABLE III. Multi-state secondary structure conditioning

sequence	condition
parent N-term	`XHHHHHHHHHHHHHLLLHHHHHHHHHHHHHLLLEEEEEELLLEEEEEXXXXXX`
child 1	`XHHHHHHHHHHHHHLLLHHHHHHHHHHHHHLLLHHHHHHHHHHHHHHHHHHHX`
parent C-term	`XEEEEELLLEEEEEEELLLHHHHHHHHHHHHLLLHHHHHHHHHHHHHX`
child 2	`XHHHHHHHHHHHHHHLLLHHHHHHHHHHHHHLLLHHHHHHHHHHHHHX`
